# Integrated transcriptomic and metabolomic analysis reveal the mechanism of citral production in *Camphora officinarum* Nees ex Wall leaves

**DOI:** 10.3389/fpls.2025.1651615

**Published:** 2025-12-15

**Authors:** Qingyan Ling, Beihong Zhang, Junfei Jiang, Zufei Xiao, Zhipeng Zhu, Lina Huang, Yuanqiu Liu, Zhinong Jin

**Affiliations:** 1 Jiangxi Provincial Engineering Research Center for Seed-Breeding and Utilization of Camphor Trees, School of Soil and Water Conservation, Jiangxi University of Water Resources and Electric Power, Nanchang, China; 2 Jiangxi Key Laboratory of Subtropical Forest Resources Cultivation, College of Forestry, Jiangxi Agricultural University, Nanchang, China

**Keywords:** *Camphora officinarum* Nees ex Wall, transcriptomic, metabolomic, citral, monoterpene

## Abstract

*Camphora officinarum* Nees ex Wall (*C. officinarum*), a citral-rich aromatic plant, is recognized as an unrivalled natural source of citral for spice production. Its large-scale cultivation in China via the sustainable coppice-rotation system has markedly revitalized rural economies. Nevertheless, the genetic basis driving its prolific citral accumulation remains elusive. We conducted an integrated transcriptomic and metabolomic analysis comparing three citral type accessions (C1, C2, C3) with a non-citral type control (C0). Metabolomic profiling identified 904 leaf metabolites, with terpenoids representing the most abundant class (19.49%). Strikingly, GC-MS analysis unveiled a monoterpene-dominated essential oil composition in citral type *C. officinarum leaves*, characterized by four dominant constituents: geranial (36.9%-44.7%), neral (30.7%-34.1%), E-isocitral (2.2%-2.9%), and Z-isocitral (1.5%-2.1%). Integrated transcriptomic and metabolomic analysis highlighted critical key genes, acetyl-CoA C-acetyltransferase (*CoAACT*), hydroxymethylglutaryl-CoA synthase (*CoHMGS)*, hydroxymethylglutaryl-CoA reductase (*CoHMGR*), phosphomevalonate decarboxylase (*CoMVD*), 1-deoxy-D-xylulose-5-phosphate synthase (*CoDXS*), geranylgeranyl diphosphate synthase, type Ⅲ (*CoGGPS)*, farnesyl diphosphate synthase (*CoFDPS*) showed elevated expression, enhancing precursor availability. The geraniol synthase (*CoGES*) and alcohol dehydrogenase (*CoADH*) involved in citral synthesis were significantly up-regulated in citral type *C. officinarum*. These findings demonstrate that the quantitative disparities in terpenoid distribution and concentration collectively define the species’ unique aromatic identity, underscoring chemotype-specific metabolic regulation mechanisms, while also screening key genetic determinants of citral biosynthesis preliminarily, thereby laying the groundwork for precision breeding programs in aromatic *C. officinarum*.

## 1 Introduction

Citral (3,7-dimethyl-2,6-octadienal), a monoterpenoid compound, exists as a mixture of two geometric isomers, geranial and neral. It serves as a key chemical precursor in the synthesis of vitamin A, vitamin E, menthol, and the high-value fragrance ingredient α-lonone (Hirai et al., 2022), Furthermore, citral exhibits a range of bioactive properties, including anti-inflammatory, antimicrobial, antioxidant, and insecticidal activities (Ling et al., 2022; Luo et al., 2022; Silva et al., 2022), which underpins its widespread use in the food, pharmaceutical, and cosmetic industries. Currently, global demand for citral has shown steady growth, with its market price substantially exceeding that of comparable terpenoids such as linalool and camphor. Commercial citral production relies on two primary approaches: chemical synthesis and plant extraction. However, the former faces environmental challenges due to high-concentration wastewater generation, whereas plant-derived natural citral aligns with consumer preferences for green and sustainable products, demonstrating significant commercial potential (Southwell, 2021). Nevertheless, limited availability of citral-rich plant resources and low essential oil content have spurred increasing research interest in citral-producing aromatic species worldwide. Internationally, natural citral is primarily sourced from *Backhousia citriodora* (Southwell et al., 2000; Southwell, 2021; Hamzah et al., 2025), *Cymbopogon citratus* (Mukarram et al., 2022), and *Ocimum gratissimum* (Kumar et al., 2021). In China, citral production predominantly depends on the fruits of *Litsea cubeba* (Fan et al., 2023), yet challenges such as a narrow harvesting window, predominantly wild-growing populations, and high harvesting costs have resulted in chronic domestic supply shortages.

*Camphora officinarum* (*C. officinarum*) exhibits chemical polymorphism (Hou et al., 2025), and its oil demonstrates strong antioxidant properties, making it widely used in the food and cosmetic industries (Zhao et al., 2025). Through preliminary efforts to collect, document, and preserve over 100-year-old germplasms from 196 provenances within its natural distribution range in China (Zhang et al., 2025a), we identified the citral-rich as a valuable chemotype among its variants. Citral content varies significantly among different citral type *C. officinarum* varieties, ranging from 52.51% to 78.8%. This substantial variability results in inconsistent quality of currently cultivated citral type aromatic camphor trees, severely hindering industry advancement (Ling et al., 2022; Hou et al., 2024). Therefore, developing high-quality, high-essential oil content varieties has become a critical objective for *C. officinarum* breeding programs. However, conventional hybridization approaches face limitations such as small floral structures, prolonged breeding cycles, and low hybridization efficiency (Liu et al., 2024). Molecular breeding, leveraging genomic insights for targeted germplasm innovation, has emerged as a strategic priority to overcome these constraints and drive sustainable industry development. Many previous molecular studies on *C. officinarum* could only be predicted by comparing the genomes of the same family species, such as *Camphora kanahirae* and *L. cubeba*, which led to insufficient identification of *C. officinarum* core functional genes (Jiang et al., 2014; Chen et al., 2018). As a result, existing data are inadequate to guide consistent cultivation and breeding of varieties with high biomass, high essential oil content and high citral content. Concurrently, the scarcity of wild citral type *C. officinarum* populations necessitates comprehensive investigation into both its extant genetic reservoirs and the molecular mechanisms underlying its essential oil biosynthesis.

Plant transcriptomic technologies have become instrumental in elucidating functional genes and characterizing key enzymes involved in secondary metabolite biosynthesis (Hou et al., 2020, Hou et al., 2023; Lu et al., 2024; Zeng et al., 2025). Concurrently, the progressive release of the *C. officinarum* genome has established a robust foundation for investigating its molecular mechanisms (Sun et al., 2021; Shen et al., 2022; Zhang et al., 2025b). As terminal phenotypic manifestations, metabolites directly reflect the dynamic interplay between genetic regulation and environmental influences (Zhang et al., 2020; Zhong et al., 2022). Through transcriptomic and metabolomic approaches, numerous monoterpene synthases and functional genes in aromatic plants and medicinal herbs have been identified (Qin et al., 2025). These include paralogous genes in the mevalonic acid (MVA) pathway—such as acetyl-CoA C-acetyltransferase (*AACT*), hydroxymethylglutaryl-CoA reductase (*HMGR*), and phosphomevalonate decarboxylase (*MVD*),—and genes in the methylerythritol phosphate (MEP) pathway, including 1-deoxy-D-xylulose-5-phosphate synthase (*DXS*), 1-deoxy-D-xylulose-5-phosphate reductoisomerase (*DXR*), 2-C-methyl-D-erythritol 4-phosphate cytidylyltransferase (*ispD)*, and 1-hydroxy-2-methyl-2-(E)-butenyl-4-diphosphate reductase (*HDR*) (Peng et al., 2025). Both MVA and MEP pathways involve terpene synthases (*TPSs*) responsible for monoterpene production, with monoterpene synthases (*mTPSs*) serving as critical rate-limiting enzymes (Xi et al., 2025). The remarkable diversity of terpenoid compounds in plants primarily stems from *TPSs* expansion and tandem duplication (Tu et al., 2025). In Lauraceae species, the number of *mTPSs* has significantly increased. For instance, *C. officinarum* contains 83 *mTPSs* (Shen et al., 2022), *C. kanehirae* has 101 (Chaw et al., 2019), and *L. cubeba* possesses 54 (Chen et al., 2020). Current research on terpenoid biosynthesis and skeletal modification in Lauraceae species confirms that chemotype formation correlates with genetic factors (Ma et al., 2025). The *CpTPS1*, *CpTPS3*, *CpTPS4* were associated with camphor, 1,8-cineole and linalool biosynthesis in *Cinnamomum porrectum* through RNA-Seq analysis of different chemotypes (Qiu et al., 2019). The *CcTPS16* and *CcTPS54* were responsible for 1,8-cineole and nerolidol synthesis in *C. officinarum* (Wang et al., 2022b). And *LcTPS42* was a key gene regulating citral biosynthesis in *L. cubeba* (Chen et al., 2020). Using microbial fermentation mediated by the borneol synthase *CbTPS1* from *Cinnamomum burmanni*, (+)-borneol was biosynthesized (Ma et al., 2021a). In the same year, the research team identified the borneol dehydrogenase *CcBDH3* in *C. officinarum*, which catalyzes the NAD^+^-dependent oxidation of (+)-borneol to (+)-camphor (Ma et al., 2021b). However, the molecular mechanism of citral metabolism in *C. officinarum* remains unexplored.

Therefore, this study takes citral-type *C. officinarum* as the research object, with non-citral type *C. officinarum* as the control, aiming to elucidate the molecular mechanism of citral biosynthesis in *C. officinarum* through integrated transcriptomic and metabolomic analyses. The specific research objectives include: (1) screening germplasms of *C. officinarum* with varying citral contents and analyzing their essential oil composition and metabolic profiles; (2) identifying key candidate genes involved in the MVA and MEP pathways as well as the terpene synthase; (3) mining genes regulating citral synthesis to provide a theoretical basis and technical support for molecular-assisted breeding in *C. officinarum*. The findings will fill the molecular knowledge gap in the citral biosynthetic pathway in *C. officinarum*, promote the breeding of high-yielding varieties, and alleviate the shortage of natural citral supply.

## 2 Materials and methods

### 2.1 Plant materials and processing

The experiment was carried out in the cutting orchard of Jiangxi provincial engineering research center for seed-breeding and utilization of camphor trees of Nanchang institute of technology (latitude: 28°41’47’N, longitude: 116°1’49’E), with a total area of 12,000 m^2^. The experimental area was a subtropical monsoon climate, with an average air temperature of 19.6°C in 2022, an annual precipitation of 1,558.9 mm, and an average of 1,816.5 h of sunshine per annum. The soil type of the test site was the fourth red clay soil. 4-year-old cuttings of three varieties of citral type *C. officinarum* (C1, C2, C3) and one variety of non-citral type *C. officinarum* (C0) growing in the same environment were randomly collected in July 2022 (**Supplementary Table S1**). All experimental plants were confirmed to be diploid.

Mature leaves were aseptically collected from six individual plants. Sampling was conducted systematically by harvesting 10 g of healthy, pest-free foliage from the eastern, southern, western, and northern sectors of each plant. The leaves were flash-frozen in liquid nitrogen for 15 min and subsequently stored at -80°C for transcriptome sequencing and leaves volatile metabolome analysis. Additionally, another 200 g of leaf samples were collected for essential oil extraction. The composition of the essential oil was determined using gas chromatography–mass spectrometry (GC–MS), with all samples analyzed in three biological replicates.

### 2.2 Measurement indicators and methods

#### 2.2.1 Essential oil extraction and quantification

Immediately after collection, 200 g leaves were taken in a 1500 mL essential oil stem distilation equipment (No. 201710158988.8) for extraction of essential oil for 90 min. The essential oils were dried over a small amount of anhydrous sodium sulphate, weighed and stored under refrigeration (4 °C) for gas chromatography-mass spectrometry (GC-MS) analysis of essential oil composition. GC-MS analysis was conducted using an electron ionization (EI) source operated at 70 eV. The temperatures of the ion source, transfer line, and quadrupole were maintained at 230°C, 250°C, and 150°C, respectively. Full-scan mass spectra were acquired over a range of m/z 50–650 with a multiplier voltage of 1200 V. The separation was performed on a capillary column (30 m × 250 μm × 0.25 μm) with high-purity helium as the carrier gas at a constant flow rate of 1.0 mL/min. The oven temperature program was set as follows: held at 80°C for 5 min, ramped at 2.5°C/min to 120°C (held for 1 min), then increased at 20°C/min to 240 °C (held for 8 min). Injection was carried out at 100°C in split mode (20:1) with a volume of 1 μL and a solvent delay of 3 min. Quantification was achieved via the area normalization method. Citral was identified by comparing its data with an authentic standard purchased from Aladdin. For further compound identification, a C7–C40 n-alkane standard mixture was co-injected to calculate the Retention Index (RI) for each component. These RIs were then used to identify compounds by matching them against reference data in the NIST library (https://webbook.nist.gov/chemistry/) (Ling et al., 2024).


$$\text{RI }=\;100[\frac{\log_{10}{\text{X}}_{\text{i}}-\log_{10}{\text{X}}_{\text{n}}}{\log_{10}{\text{X}}_{\text{n}+1}-\log_{10}{\text{X}}_{\text{n}}}+\text{n}]$$

where X_i_, X_n_, and X_n+1_ represent the retention times of the target metabolite, the n-alkane eluting immediately before it, and the n-alkane eluting immediately after it, respectively; and n is the carbon number of the n-alkane prior to the metabolite.

At the same time, six leaves were randomly taken to measure the water content by MA150 Rapid Moisture Analyzer (Sartorius, Germany), which was repeated three times to calculate the oil essential oil content (%).


$$\text{Yf}(\%)=100\times \frac{M_{1}}{M_{2}}$$

$$\text{Yd}(\%)=\frac{100\times M_{1}}{(1-W)\times M_{\begin{array}{c} 2 \end{array}}}$$

Where Yf was the essential oil content of fresh leaves; Yd was the essential oil content of dry leaves; M1 was the weight of the extracted essential oil; M2 was the weight of fresh leaves; and W was the water content.

#### 2.2.2 SPME analysis of leaf volatile compounds

Five grams of C0, C1, C2, and C3 *C. officinarum* leaves samples were randomly collected with three biological replicates per group, weighed, and immediately flash-frozen in liquid nitrogen to preserve biochemical integrity. Subsequently, the frozen tissues were homogenized into a fine powder using a cryogenic grinder. Immediately upon opening, 500 mg (approximately 1 mL) of the powder was weighed into a 20 mL headspace vial (Agilent, Palo Alto, CA, USA) containing a saturated NaCl solution to suppress enzymatic activity. The vial was sealed using a crimp-top cap with a polytetrafluoroethylene (TFE)-silicone septum (Agilent). For SPME extraction, each vial was equilibrated at 60°C for 5 min, after which a 120 µm DVB/CWR/PDMS fiber (Agilent) was exposed to the headspace for 15 min at the same temperature. Following extraction, volatile organic compounds (VOCs) were desorbed from the fiber in the injection port of an Agilent 8890 GC system at 250°C for 5 min in splitless mode. VOC separation was performed using the same GC system coupled to a 7000D mass spectrometer (Agilent), equipped with a DB-5MS capillary column (30 m × 0.25 mm × 0.25 µm). High-purity helium was used as the carrier gas at a constant linear velocity of 1.2 mL/min. The injector and transfer line temperatures were maintained at 250°C and 280°C, respectively. The oven temperature program was as follows: held at 40°C for 3.5 min, increased to 100°C at 10°C/min, then to 180°C at 7°C/min, and finally to 280°C at 25°C/min, with a final hold time of 5 min. Mass spectrometric detection was conducted in electron ionization (EI) mode at 70 eV. The ion source and quadrupole temperatures were set to 230°C and 150°C, respectively. Data acquisition was performed in selected ion monitoring (SIM) mode for the identification and quantification of target analytes. Determination, identification and quantification of volatile organic compounds were referred to the literature (Wei et al., 2016), and the differential metabolites DAMs were determined based on VIP ≥ 1 and |Log2FC| ≥ 1.0.

#### 2.2.3 RNA sequencing and data analysis

Leaf tissue preserved at -80 °C was pulverized into fine powder in liquid nitrogen, and total RNA was extracted using the RNAprep Pure Plant Plus Kit (Tiangen, DP441). Total RNA was extracted and quantified. RNA integrity was detected by Agilent 2100 Bioanalyser, and OD260/OD280 of each sample was in the range of 1.8 - 2.2, OD260/OD230≧1.5, RNA molecular integrity index (RIN)≧6.5, rRNA 28S/18S≧1.0, RNA concentration should be no less than 400 ng/μL, and the total amount of RNA should be no less than 20 μg. The library was constructed using the Illumina NEBNext^®^ Ultra™ RNA Library Prep Kit. Briefly, poly(A)-tailed mRNA was enriched using Oligo(dT) magnetic beads. The purified mRNA was then randomly fragmented in NEB Fragmentation Buffer via divalent cations. Using the fragmented mRNA as template, first-strand cDNA was synthesized with random oligonucleotide primers in an M-MuLV Reverse Transcriptase system. The RNA strand was subsequently degraded by RNase H, and second-strand cDNA was synthesized using DNA Polymerase I with dNTPs as substrates. The purified double-stranded cDNA underwent end repair, was adenylated at the 3’ end, and was ligated to sequencing adapters. cDNA fragments of approximately 200 bp were selected using AMPure XP beads, followed by PCR amplification. The PCR product was further purified with AMPure XP beads to generate the final library. Following construction, the library was initially quantified using a Qubit 2.0 Fluorometer and diluted to 1.5 ng/μL. The insert size was then assessed using an Agilent 2100 Bioanalyzer. Upon confirmation that the insert size met expectations, the library’s effective concentration was accurately quantified via qRT-PCR (required to be >2 nM) to ensure quality. Finally, qualified libraries were sequenced on the Illumina NovaSeq 6000 platform. The reference genome used was *C. officinarum* reference genome (GWHBGBX00000000.genome.fasta.gz), downloaded from (https://ngdc.cncb.ac.cn/gwh/Assembly/23429/show).

#### 2.2.4 Gene function annotation and differential gene screening

All Unigenes were compared and functionally annotated with the non-redundant protein sequences NR, Pfam, Swissprot, Gene Ontology GO, Kyoto Encyclopaedia of Genes and Genomes KEGG, Trembl, KOG, and Transcription Factor Database TF, and the PFAM protein annotations were performed with the software Hmmscan (HMMER3).

The transcript expression level was measured based on the gene length per million mapped reads per kilobase (FPKM). Differential expression analysis was performed using DESeq2 (v1.22.1), and the P-value was corrected using the Benjamini & Hochberg method to obtain the Q-value, and the Unigenes with multiplicity of difference |log2foldchange|>1 and Q-value < 0.05 were differentially expressed genes (DEGs). The raw sequence data has been submitted to the NCBI (PRJNA1132669).

#### 2.2.5 qRT-PCR validation of differentially expressed genes

Gene-specific primers were designed using Primer3 software (**Supplementary Table S4**) and validated through qRT-PCR with SYBR Green PCR Master Mix. The thermal cycling protocol consisted of an initial denaturation at 95°C for 5 minutes, followed by 40 cycles of denaturation at 95°C for 10 s and annealing/extension at 60°C for 30 s. Post-amplification melting curve analysis was performed by gradual temperature elevation from 60°C to 95°C with 0.5°C increments every 10 s to confirm primer specificity. The Actin gene was used as the endogenous control, and the gene expression levels were quantified in triplicate biological samples using the comparative 2^-△△ct^ method (Livak and Schmittgen, 2001).

The Pearson correlation coefficient was calculated using SPSS software to assess the correlation between qRT-PCR expression levels and transcriptomic data in citral type and non-citral type *C. officinarum*. The formula is as follows:


$$r=\frac{\sum_{i=1}^{n}(x_{i}-\bar{x})(y_{i}-\bar{y})}{\sqrt{\sum_{i-1}^{n}{\left(x_{i}-\bar{x}\right)}^{2}}\sqrt{\sum_{i-1}^{n}{\left(y_{i}-\bar{y}\right)}^{2}}}$$

where: *x* and *y* represent qRT-PCR expression values and transcriptomic data values, respectively n denotes the number of samples.

The Pearson correlation coefficient (*r*) ranges from -1 to 1. Values approaching 1 or -1 indicate strong linear relationships, while values near 0 suggest no linear correlation. A positive *r* signifies a positive correlation, whereas a negative *r* reflects an inverse relationship.

### 2.3 Statistical analysis

The statistical analyses were conducted using SPSS 26.0 software. Data were reported as means ± standard deviation of three replicates. Differences were tested for significance by using the ANOVA procedure, using a significance level of *p ≤*0.05. Duncan’s multiple range test at probability level 5% was used to compare the mean of data. The essential oil components were analyzed using mass spectrometry quantitative qualitative Masshunter software. Other analysis charts were drawn by Origin 2023b software. In the correlation analysis between genes and metabolites, the Pearson correlation coefficients were calculated using the cor function in R4.2.0. Gene–metabolite pairs with a correlation coefficient greater than 0.90 and *p*-value less than 0.01 were selected for downstream analysis.

## 3 Results

### 3.1 Leaf morphology and essential oil indexes

Comparative analysis revealed distinct morphological variations among the treatment groups: C1 C2 and C3 exhibited 9.4%, 7.8% and 4.5% greater leaf lengths respectively compared to C0 (*p* < 0.05), while C3 demonstrated a 7.3% reduction in leaf width relative to C0 (*p* < 0.05). The difference in leaf area of citral type C1, C2, C3 and non-citral type *C. officinarum* C0 was not significant. The essential oil contents of citral type *C. officinarum* leaves were 0.93%-1.02% (FW, w/w) and 2.20%-2.42% (DW, w/w), which were significantly smaller than that of non-citral type *C. officinarum* leaves of 1.66% (FW, w/w) and 3.25% (DW, w/w) (*p* ≤ 0.05). However, the difference in essential oil content of citral type leaves C1, C2, and C3 was not significant (**Table 1**).

**Table 1:** Leaf morphology index and essential oils content of the different chemotypes of C. *officinarum*.

Different varieties	C1	C2	C3	C0
leaf length (mm)	98.0 ± 2.92^a^	96.6 ± 2.44^a^	93.6 ± 1.91^ab^	89.6 ± 0.93^b^
leaf width (mm)	49.82 ± 2.75^a^	47.04 ± 0.68^ab^	43.9 ± 1.24^c^	47.34 ± 1.1^ab^
leaf area (mm^2^)	3064.88 ± 275.11^a^	2766.26 ± 72.07^a^	2599.04 ± 219.62^a^	2681.16 ± 75.66^a^
Fresh weight EOs content (%)	1.01 ± 0.06^b^	0.93 ± 0.08^b^	1.02 ± 0.09^b^	1.66 ± 0.17^a^
Dry weight EOs content (%)	2.37 ± 0.13^b^	2.20 ± 0.21^b^	2.42 ± 0.22^b^	3.25 ± 0.35^a^

Different lowercase letters in the same line indicate significant differences between different varieties of *C. officinarum (p* < 0.05).

A total of 39 compounds, including 11 monoterpenes, 26 sesquiterpenes, and 2 heterocyclic compounds, were identified from citral type (C1, C2, and C3) and non-citral type (C0) *C. officinarum* leaf essential oils (**Table 2**). The monoterpenes and oxygenated monoterpenes accounted for 75.4% to 86.1%. In the essential oils of citral type *C. officinarum*, the isomeric pair neral and geranial emerged as dominant constituents, accounting for 69.5%-78.8% of the total volatile profile based on chromatographic quantification. Among the non-citral type *C. officinarum* (C0), linalool (84.2%) was the highest.

**Table 2:** Essential oil composition of the different chemotypes of C. *officinarum* leaves.

No	RI(lit)	RI (exp)	Compounds	Molecular formula	Percent composition(%)			
					C1	C2	C3	C0
1	934	937	α-Pinene	C_10_H_16_	0.75 ± 0.03	0.3 ± 0.01	0.4 ± 0.01	–
2	1030	1029	eucalyptol	C_10_H_18_O	–	0.42 ± 0.04	0.1 ± 0.01	–
3	1101	1098	linalool	C_10_H_18_O	–	–	–	84.2 ± 0.9
4	1144	1141	camphor	C_10_H_16_O	–	–	–	1.9 ± 0.03
5	1148	1143	citronelleal	C_10_H_18_O	0.51 ± 0.07	0.78 ± 0.06	0.53 ± 0.04	–
6	1165	1165	Z-isocitral	C_10_H_16_O	2.03 ± 0.13	1.50 ± 0.06	2.1 ± 0.06	–
7	1184	1179	E-isocitral	C_10_H_16_O	2.7 ± 0.09	2.9 ± 0.07	2.2 ± 0.06	–
8	1245	1247	neral	C_10_H_16_O	34.1 ± 0.97	30.7 ± 0.58	32.6 ± 0.48	–
9	1254	1244	geraniol	C_10_H_18_O	0.6 ± 0.04	0.5 ± 0.02	0.44 ± 0.02	–
10	1276	1277	geranial	C_10_H_16_O	44.7 ± 0.66	40.2 ± 0.59	36.9 ± 0.54	–
11	1355	1346	geranic acid	C_10_H_16_O_2_	–	0.1 ± 0.01	0.1 ± 0.02	–
12	1356	1353	β-Citronellyl acetate	C_12_H_22_O_2_	–	0.4 ± 0.01	0.6 ± 0.01	–
13	1388	1380	β-elemene	C_15_H_24_	0.89 ± 0.08	0.79 ± 0.07	0.55 ± 0.05	0.7 ± 0.01
14	1384	1383	geranyl acetate	C_12_H_20_O_2_	–	0.8 ± 0.02	4.1 ± 0.08	–
15	1410	1403	caryophyllene	C_15_H_24_	0.8 ± 0.05	1 ± 0.02	1.2 ± 0.04	1.8 ± 0.03
16	1432	1430	γ-elemene	C_15_H_24_	–	–	–	5.6 ± 0.11
17	1454	1454	humulene	C_15_H_24_	0.75 ± 0.07	1 ± 0.02	1.3 ± 0.02	1 ± 0.02
18	1480	1477	germacrene D	C_15_H_24_	–	0.3 ± 0.01	0.2 ± 0.0	0.4 ± 0.01
19	1486	1484	β-selinene	C_15_H_24_	0.12 ± 0.03	0.6 ± 0.01	0.4 ± 0.01	–
20	1495	1491	bicyclogermacrene	C_15_H_24_	0.6 ± 0.04	0.1 ± 0.03	0.8 ± 0.01	–
21	1524	1518	delta-cadinene	C_15_H_24_	0.27 ± 0.04	0.35 ± 0.07	0.2 ± 0.03	–
22	1537	1540	elemol	C_15_H_26_O	0.5 ± 0.01	0.4 ± 0.01	0.4 ± 0.01	–
23	1562	1557	(E)-nerolidol	C_15_H_26_O	0.5 ± 0.02	0.6 ± 0.02	0.3 ± 0.03	0.2 ± 0.01
24	1577	1571	10’-apocarotenal	C_15_H_24_O	0.6 ± 0.01	0.9 ± 0.01	3.9 ± 0.06	–
25	1576	1572	spathulenol	C_15_H_24_O	0.20 ± 0.04	0.3 ± 0.01	0.5 ± 0.01	0.7 ± 0.01
26	1578	1574	caryophyllene oxide	C_15_H_24_O	0.8 ± 0.01	1.1 ± 0.02	–	0.2 ± 0.05
27	1580	1579	globulol	C_15_H_26_O	0.98 ± 0.05	–	–	0.2 ± 0.06
28	1584	1581	viridiflorol	C_15_H_26_O	–	–	–	0.1 ± 0.04
29	1591	1587	guaiol	C_15_H_26_O	0.4 ± 0.01	0.3 ± 0.01	0.56 ± 0.10	–
30	1593	1589	humulene oxide I	C_15_H_24_O	0.45 ± 0.09	1.21 ± 0.08	0.3 ± 0.01	–
31	1631	1632	humulene II	C_15_H_24_O	0.23 ± 0.06	1.4 ± 0.03	1.5 ± 0.03	0.2 ± 0.01
32	1660	1662	neointermedeol	C_15_H_26_O	0.84 ± 0.07	0.78 ± 0.06	0.1 ± 0.01	0.2 ± 0.03
33	1669	1672	alloaromadendrene oxide-(1)	C_15_H_24_O	0.49 ± 0.05	0.43 ± 0.11	0.52 ± 0.09	–
34	1689	1694	(1R,7S,E)-7-Isopropyl-4,10-dimethylenecyclodec-5-enol	C_15_H_24_O	0.42 ± 0.14	0.45 ± 0.09	–	–
35	1706	1705	(Z, Z)-farnesol	C_15_H_26_O	0.26 ± 0.03	0.2 ± 0.02	0.2 ± 0.02	0.1 ± 0.01
36	1713	1714	6-Isopropenyl-4,8a-dimethyl-1,2,3,5,6,7,8,8a-octahydro-naphthalen-2-ol	C_15_H_24_O	0.31 ± 0.07	1.24 ± 0.08	1.38 ± 0.11	–
37	1719	1718	E, E-Farnesal	C_15_H_24_O	–	1.36 ± 0.12	0.15 ± 0.04	–
38	1805	1802	14-OH-δ-Cadinene	C_15_H_24_O	0.74 ± 0.08	0.44 ± 0.07	1.58 ± 0.09	–
39	1822	1809	Selina-4,7-diol	C_15_H_28_O_2_	–	0.31 ± 0.08	–	–
Amount of chemical compounds					28	34	31	15
Total identified constituents					96.54	94.16	96.11	97.5
Hydrocarbon monoterpenes (HM) 1.					0.75	0.3	0.4	0
Oxygenated monoterpenes (OM) 2-11.					84.64	77.1	74.97	86.1
Hydrocarbon sesquiterpenes (HS) 13,14-21.					3.43	4.14	4.65	9.5
Oxygenated sesquiterpenes (OS) 22-39.					7.72	11.42	11.39	1.9
Non terpenic compounds (NT) 12,14.					0	1.2	4.7	0

More than 0.1% are listed in the table. - not detected.

### 3.2 Metabolome analysis

The volatile metabolite correlation analysis of citral type (C1, C2 and C3) and non-citral type (C0) *C. officinarum* leaves showed a strong intra-group correlation of 0.91 for citral type *C. officinarum*, while the correlation coefficients for citral and non-citral were at or below 0.09, with significant inter-group variation (**Supplementary Figure S1a).**

The principal component analysis PC1 (49.87%) × PC2 (26.31%) showed that citral type C1, C2, C3 and non-citral type C0 were clearly separated (**Supplementary Figure S1b**), indicating that there are differences in the accumulation of secondary metabolites in different chemotypes of *C. officinarum* at the metabolic level, and that the three replicates of each variety were clustered together with good homogeneity and high reliability of data. The citral type (C1, C2, and C3) and non-citral type (C0) *C. officinarum* OPLS-DA plots showed Q^2^ of 0.999, 1, and 0.999, respectively, R^2^Y of 1 for all, R^2^X of 0.944, 0.949, and 0.956, respectively, and p-values of 0.005 for both Q^2^ and R^2^Y, which suggested that the model was eligible (**Supplementary Figure S2a, b, c**). Comprehensive profiling of the foliar volatile metabolome identified 904 volatile organic compounds (VOCs), categorized into 16 chemical classes. Quantitative analysis revealed the following predominant groups: terpenoids (19.49%), heterocyclic compounds (16.72%), esters (15.17%), ketones (8.42%), alcohols (7.86%), hydrocarbons (7.42%), aromatics (6.2%), aldehydes (5.65%), amines (3.43%), acids (3.43%), phenols (2.44%), sulphur compounds (1.44%), nitrogen compounds (1%), ethers (0.44%), halogenated hydrocarbons (0.22%), and other compounds (0.66%) (**Supplementary Figure S2d**).

**Figure 1:**
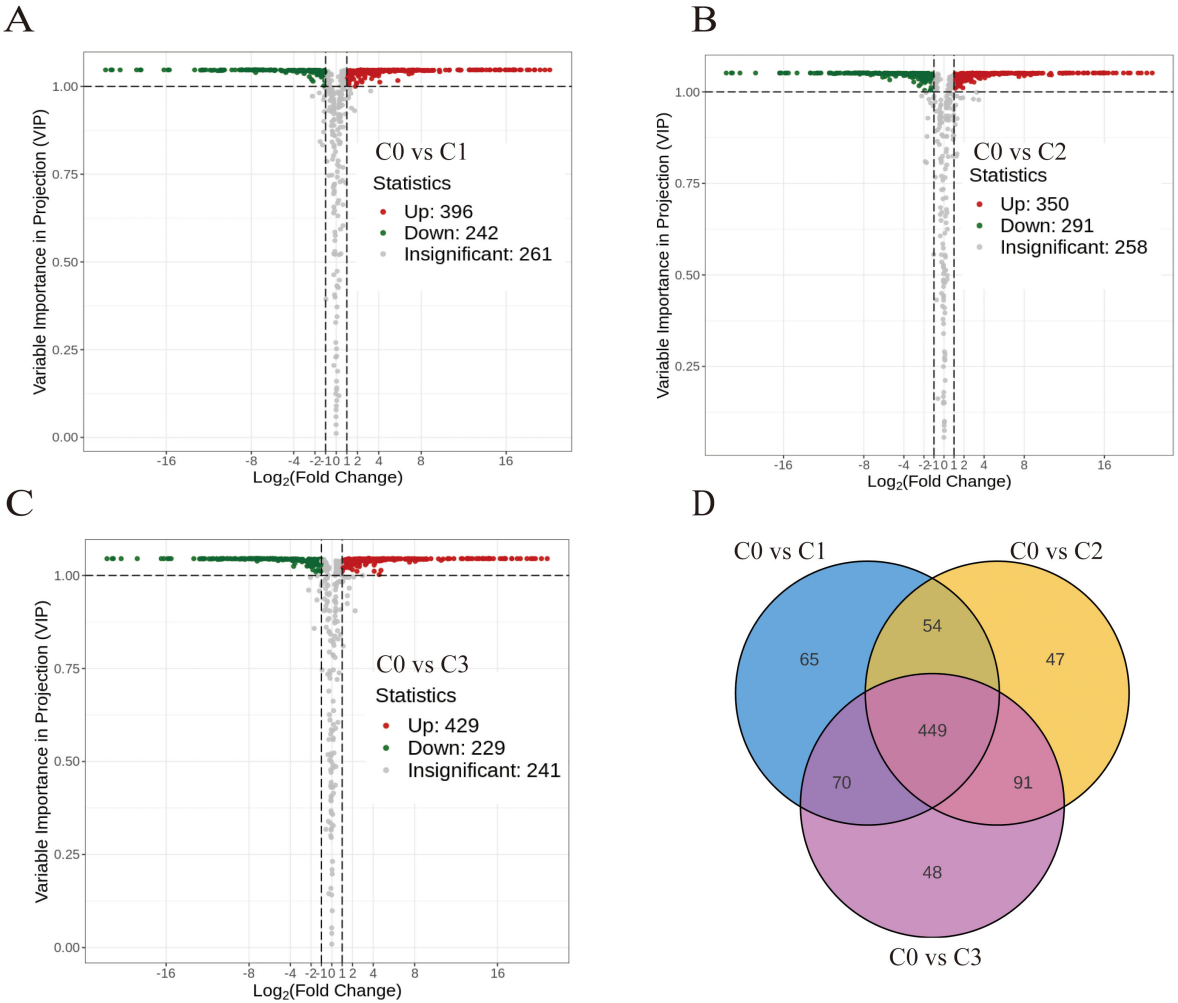
Analysis of differential metabolites between citral type and non-citral type *C. officinarum*. **(A)** C0 vs C1 differential accumulated metabolites (DAMs) volcano plot. **(B)** C0 vs C2 DAMs volcano plot. **(C)** C0 vs C3 DAMs volcano plot. **(D)** Differential metabolite Venn diagram. Note: Volcanic dots represent metabolites, where green represents down-regulation, red represents up-regulation, and grey represents no significant difference (the same below); The horizontal coordinate indicates the logarithm value of the difference multiple (log_2_FC), and the greater the absolute value of the horizontal coordinate, the greater the relative content difference. The ordinate represents -log_10_*P*, and the size of the dot represents the VIP value.

638 differentially accumulated metabolites (DAMs) for C0 vs C1, 641 DAMs for C0 vs C2, and 658 DAMs for C0 vs C3 were identified by multivariate analysis using combined thresholds of absolute fold change ≥2 (*p* < 0.05) and VIP score ≥1 from OPLS-DA modelling (**Supplementary Table S7**). The non-citral type C0 and citral type C1, C2 and C3 had a total of 449 DAMs (**Figure 1**).

Citral type *C. officinarum* C1, C2, and C3 detected 876, 872, and 866 metabolites, respectively, and different chemotypes of *C*. *officinarum* leaves had different metabolites, which was consistent with the chemotypic profile of leaf essential oils and their characteristic aroma attributes, suggesting that different terpene compositions were the main reasons for the differences in the aroma of the different chemotypes of *C. officinarum* leaves (**Table 3**).

**Table 3:** The first 10 metabolites in leaves of the *C. officinarum*.

Species	Formula	Compounds	Class I
C1、C2、C3	C_10_H_16_O	geranial	Terpenoids
	C_11_H_20_O_2_	6-octen-1-ol, 3,7-dimethyl- formate	Terpenoids
	C_7_H_7_NO_2_	1H-pyrrole-2,5-dione, 3-ethenyl-4-methyl	Heterocyclic
	C_10_H_18_O	2,4-decadien-1-ol	Alcohol
	C_10_H_14_O_2_	carvone oxide, trans-	Terpenoids
	C_15_H_24_	humulene	Terpenoids
	C_10_H_16_O	neral	Terpenoids
	C_15_H_24_	trans-caryophyllene	Terpenoids
	C_14_H_24_O_2_	1,5-dimethyl-1-vinyl-4-hexenyl butyrate	Ester
	C_15_H_24_	β- Cedrene	Terpenoids
C0	C_10_H_18_O	linalool	Terpenoids
	C_10_H_16_O	hotrienol	Alcohol
	C_11_H_18_	cyclohexene, 2,4-dimethyl-1-(1-methylethenyl)	Hydrocarbons
	C_10_H_14_O	benzene, (1-methoxypropyl)	Aromatics
	C_10_H_14_O	filifolone	Ketone
	C_10_H_14_O	perillene	Terpenoids
	C_9_H_18_O	1-nonen-4-ol	Alcohol
	C_9_H_16_O_2_	2-butenoic acid, 2-methyl-, 2-methylpropyl ester, (E)	Ester
	C_10_H_16_O	α-pinene 2,3-oxide	Terpenoids
	C_10_H_18_O	2-isopropyl-5-methylhex-2-enal	Aldehyde

**Figure 2:**
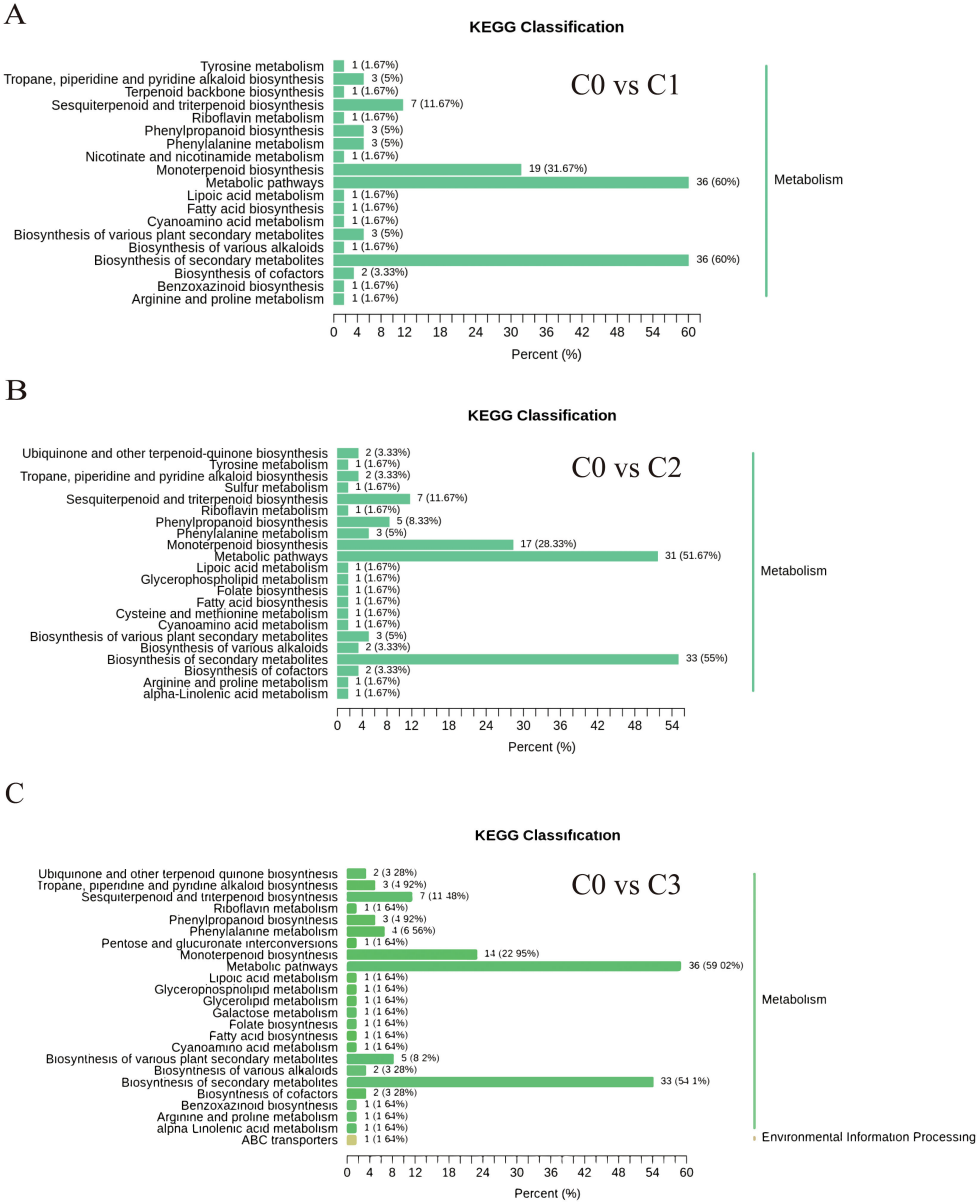
Differentially accumulated metabolites KEGG classification chart. **(A)** C0 vs C1. **(B)** C0 vs C2. **(C)** C0 vs C3.

KEGG analysis of 449 differentially accumulated metabolites (DAMs) revealed that the proportions of differential metabolites in monoterpenoid biosynthesis (ko00902), biosynthesis of secondary metabolites (ko01110), and metabolic pathways (ko01100) were relatively high, reaching 22.95%-31.67%, 51.57%-60%, and 54.1%-60%, respectively. However, the high proportion in metabolic pathways (ko01100) is attributed to its global nature (encompassing all metabolic activities), and thus the focus should be placed on the first two pathways (**Figure 2**).

Heatmap of differential metabolites of the monoterpenoid biosynthesis pathway (ko00902) and biosynthesis of secondary metabolites (ko01110) showed that the citral type *C. officinarum* C1, C2, and C3 had high content of geraniol, geranial, neral, and citronellal. Geranial and neral were the two geometric isomers of citral, and geraniol was the precursor substance for the synthesis of geranial. The highest content of linalool was found in the non-citral type *C. officinarum* C0 (**Supplementary Figure S3**).

### 3.3 Transcriptome analysis

#### 3.3.1 Quality of leaf sequencing

The RNA concentrations of C1, C2, C3 and C0 *C. officinarum* were 191–428 ng/μL, the total amount of RNA was 7.78-13.69 μg, and the completeness value was 4.40-7.60, satisfying the need to establish transcriptome libraries (**Supplementary Table S2**). The correlation coefficient of the 3 citral type varieties was greater than that between citral type and non-citral type and principal component analysis showed that citral type C1, C2, C3 and non-citral type C0 were clearly separated (**Supplementary Figure S4**). The clean data of all samples reached 6.53 Gb, Q30 was above 91.7%, and the read mapping rate of all samples was above 90.04% (**Supplementary Table S3**), indicating high assembly integrity for subsequent analysis.

#### 3.3.2 Differential gene screening

**Figure 3:**
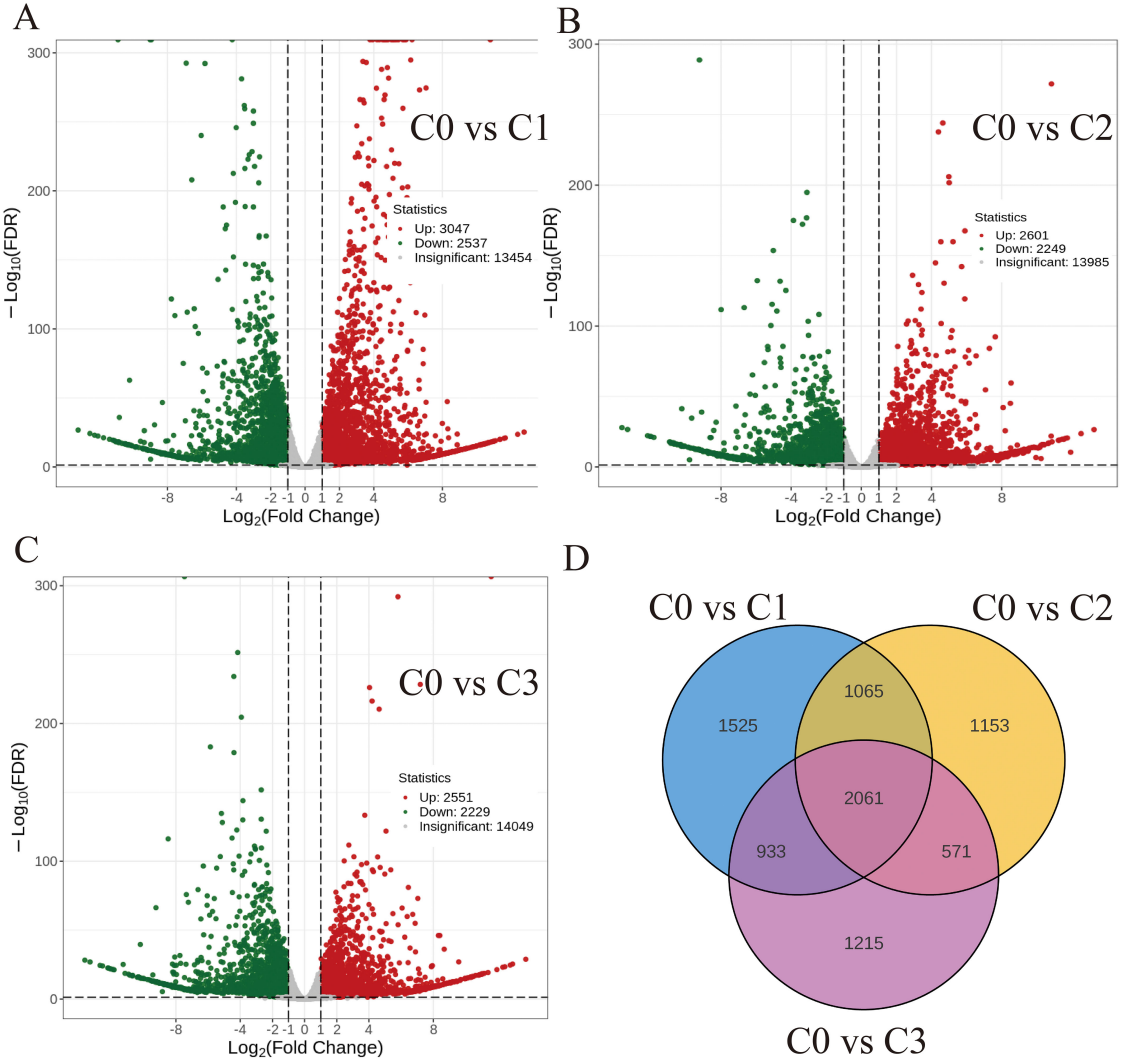
Volcano map and Venn diagram of the differentially expressed genes **(A)** C0 vs C1. **(B)** C0 vs C2. **(C)** C0 vs C3. **(D)** Differential genes Venn diagram.

Differential expression analysis (|log2FC| > 1, Q-value < 0.05) revealed 5,584 (C0 vs C1), 4,850 (C0 vs C2), and 4,780 (C0 vs C3) DEGs, with respective up-/down-regulated ratios of 3,047/2,537, 2,601/2,249, and 2,551/2,229. A core set of 2,061 DEGs was conserved across all comparisons (**Figure 3**).

GO enrichment of DEGs elucidated the DEGs function between citral type C1, C2, C3 and non-citral type C0. The 50 most significantly enriched GO terms were categorized into three functional ontologies: biological process (BP), cellular component (CC), and molecular function (MF). BP terms predominantly encompassed stress-responsive pathways, including calcium-mediated signaling, cellular recognition, and coordinated responses to chemical stimuli (acid/amino acid/hypoxia; 6 entries), organonitrogen compound processing (2 entries), immune regulation (2 entries), and phytohormone signaling (jasmonic/salicylic acid). CC terms were primarily represented by cell projection-related components (2 entries). These findings suggest that citral type *C. officinarum* monoterpene metabolism mitigates hypoxic stress through cytoarchitectural stabilization, second-messenger signaling modulation, transcriptional reprogramming, and phytohormone crosstalk (**Supplementary Figure S5**).

**Figure 4:**
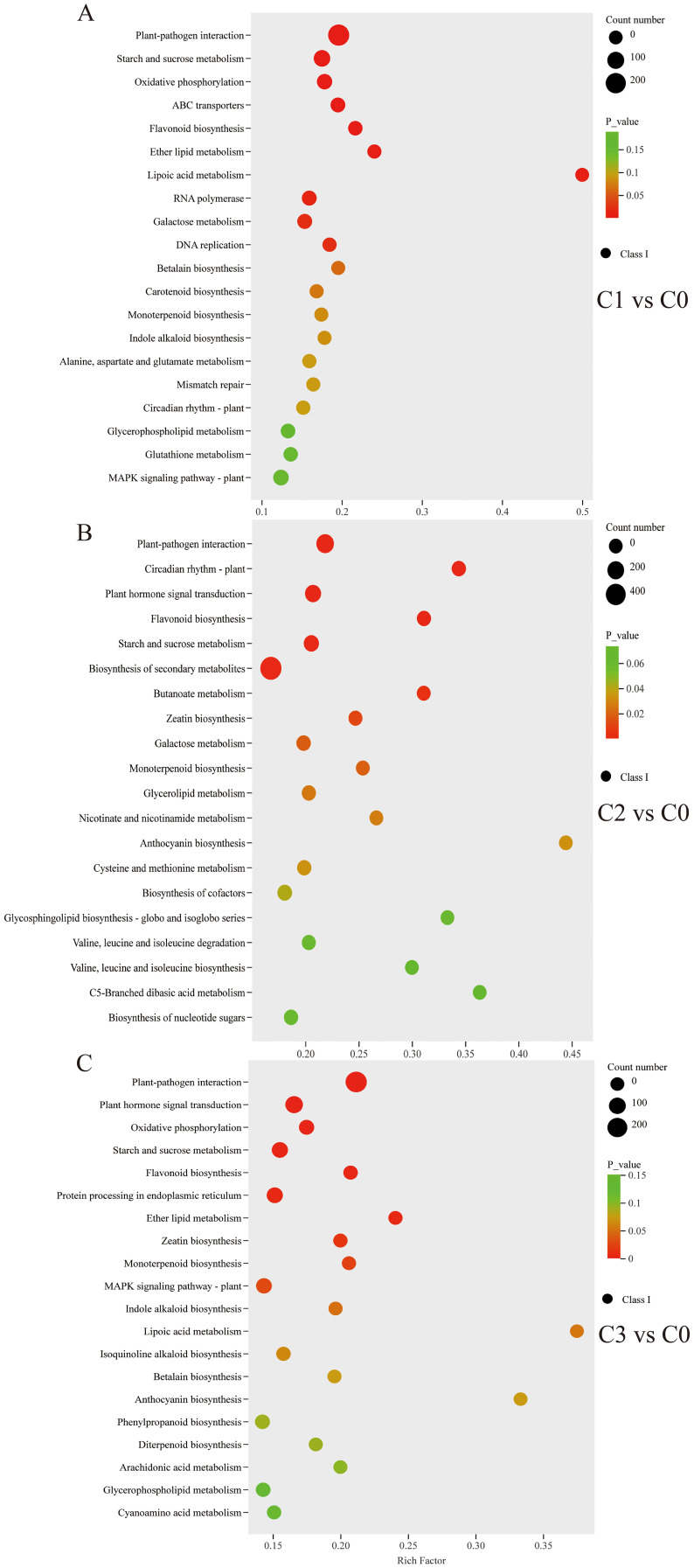
KEGG enrichment scatter plot of DEGs [**(A)** C0 Vs C1. **(B)** C0 Vs C2. **(C)** C0 Vs C3].

The top 20 metabolic pathways of DEGs obtained from the KEGG pathway enrichment analysis of C0 vs C1/C2/C3 were shown in **Figure 4**. Starch and sucrose metabolism were significantly enriched, with 54 DEGs (28 up-/26 down-regulated), 57 DEGs (32 up-/25 down-regulated), and 47 DEGs (20 up-/27 down-regulated) in the C0 vs C1/C2/C3, respectively (**Supplementary Figure S6**). In the monoterpenoid biosynthesis pathway (map00902), differential expression analysis revealed 18 DEGs (10 up-/8 down-regulated) in C0 vs C1, 16 DEGs (9 up-/7 down-regulated) in C0 vs C2, and 16 DEGs (6 up-/10 down-regulated) in C0 vs C3 comparisons (**Supplementary Figure S7**). The plant-pathogen interaction pathway (map04626) was a defence of plants in the immune response to pathogen attack. 327 DEGs (237 up-/90 down-regulated), 254 DEGs (174 up-/80 down-regulated), and 287 DEGs (193 up-/94 down-regulated) were in the C0 vs C1/C2/C3 plant-pathogen interaction pathway, respectively (**Supplementary Figure S8**).

#### 3.3.3 Combined analysis of differential genes and differential metabolites

**Figure 5:**
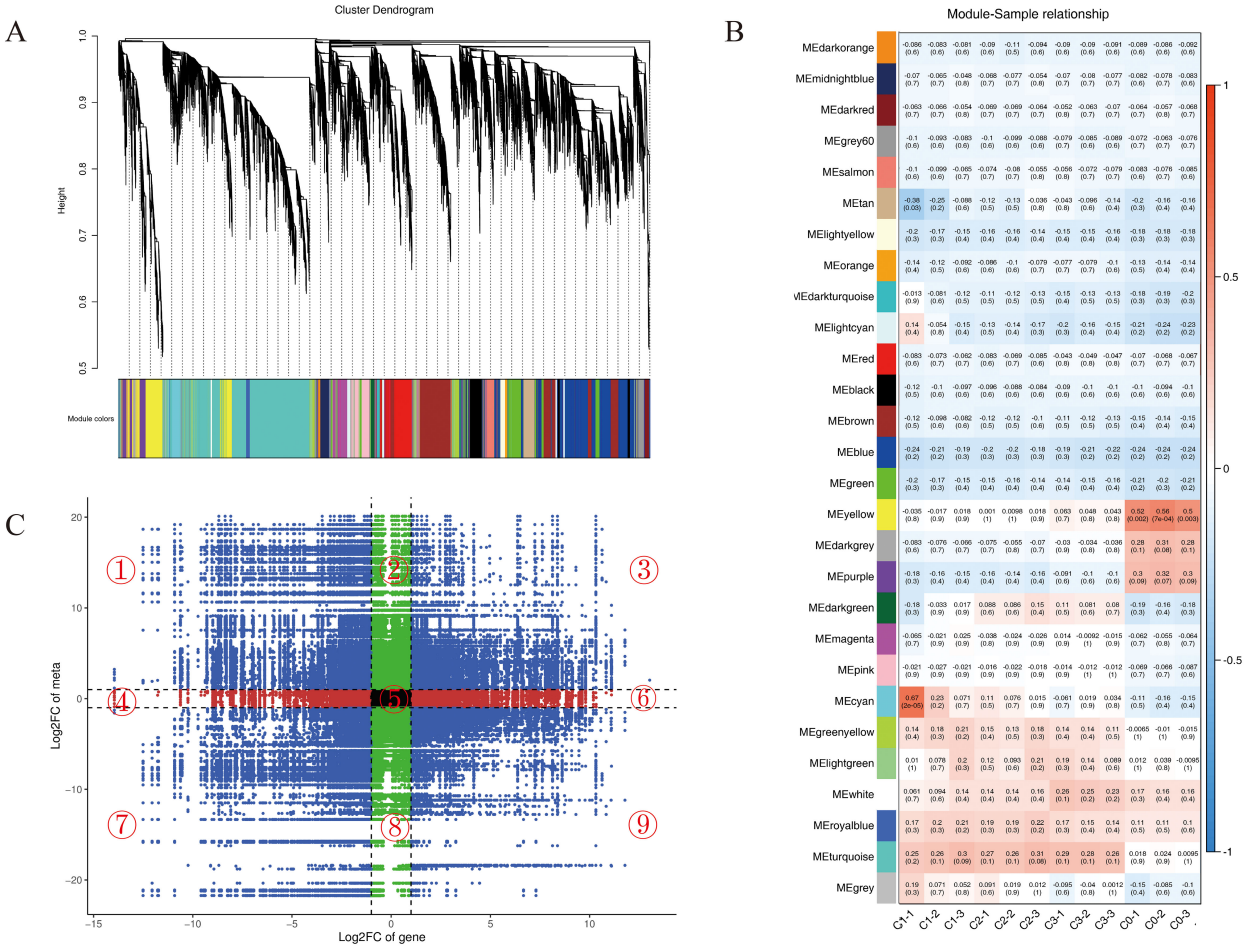
Co-expression analysis of genes and metabolites. **(A)** Cluster dendrogram of metabolite-gene co-expression modules based on WGCNA. The vertical axis represents the cluster tree height, which corresponds to the distance metric between genes, whereas the horizontal axis delineates distinct gene co-expression modules, each color-coded to a specific module. **(B)** Heat map of module-sample correlations. The numerical values within each square denote the correlation coefficients and associated *p*-values between modules and samples, with more intense colors indicating a greater absolute correlation. **(C)** Nine-quadrant plot of gene-metabolite correlation analysis. The x-axis represents the log2FC of genes from the transcriptome data, while the y-axis represents the log2FC of metabolites. The dashed line parallel to the y-axis indicates the significance threshold for transcriptomic fold change, and the dashed line parallel to the x-axis denotes the significance threshold for metabolomic fold change.

Based on transcriptome profiling data, weighted gene co-expression network analysis (WGCNA) was applied using a filtering threshold of 0.85 (excluding 85% of genes and retaining the top 15% for subsequent analysis). Hierarchical clustering analysis revealed multiple significantly associated gene co-expression modules related to the differential regulation of leaf volatile metabolites among different chemotypes of *C. officinarum* (**Figure 5A**). Module-trait correlation analysis, conducted to define the relationship between the modules and the target trait (citral type), revealed statistically significant associations: the yellow (1719 genes), darkgrey (117 genes), and purple (615 genes) modules showed significantly negative correlations with the citral type, while the cyan (467 genes), greenyellow (567 genes), lightgreen (289 genes), royalblue (220 genes), and turquoise (5515 genes) modules were significantly positively correlated with the citral type (**Figure 5B**, **Supplementary Table 5**). Based on these key modules significantly associated with chemotypes, we subsequently extracted genes from them to perform an integrated transcriptomic and metabolomic analysis. This approach was designed to move beyond the macro-level module-trait associations and directly uncover micro-level regulatory relationships between genes and metabolites.

By calculating the quantitative abundance correlations between genes and metabolites across all samples and visualizing the results via a nine-quadrant plot (**Figure 5C**), we successfully identified gene-metabolite pairs with significant putative regulatory relationships (Pearson correlation coefficient > 0.90 and p-value < 0.01). Specifically, pairs located in quadrants 3 and 7 exhibited a positive regulatory relationship, whereas those in quadrants 1 and 9 exhibited a negative relationship. This initial screening yielded tens of thousands of associated pairs. However, this initial set of associations was extensive and contextually complex. Given the primary objective of this study—to elucidate the formation mechanism of *C. officinarum* essential oil, which is predominantly composed of terpenoids (**Table 2**)—we refined our focus from these broad associations to specifically target differential genes corresponding to terpenoid metabolites. This critical filtering step resulted in a substantially reduced yet biologically more meaningful set of core association pairs (highlighted in red in **Supplementary Tables 5-6**), thereby laying a solid foundation for subsequent in-depth analysis.

**Figure 6:**
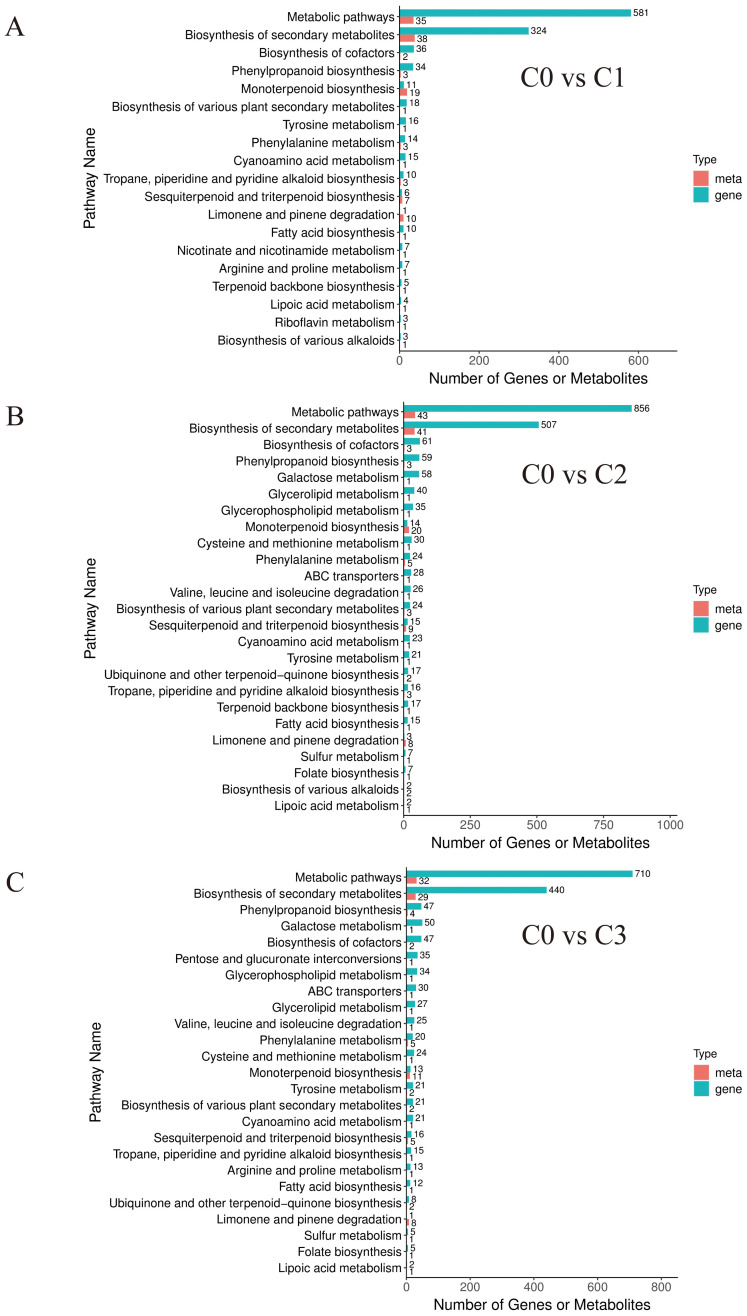
Bar chart of differential genes and differential metabolites based on combined transcriptome and metabolome analysis.

With this refined set of core gene-metabolite pairs highly relevant to terpenoid synthesis in hand, we proceeded beyond describing isolated correlations to performing pathway enrichment analysis on the integrated multi-omics data. This analysis revealed seven core conserved pathways implicated in chemotype divergence (**Figure 6**). Notably, these pathways primarily fall into three major functional categories: (1) central metabolism, (2) defense synthesis, and (3) terpenoid specialization. This pathway-level discovery strongly corroborates our earlier metabolomic findings—that the essential oil of the citral type of *C. officinarum* is predominantly enriched in monoterpenes (**Tables 2**-**3**)—and strongly suggests that terpenoid specialization pathways are central to phenotypic divergence.

**Figure 7:**
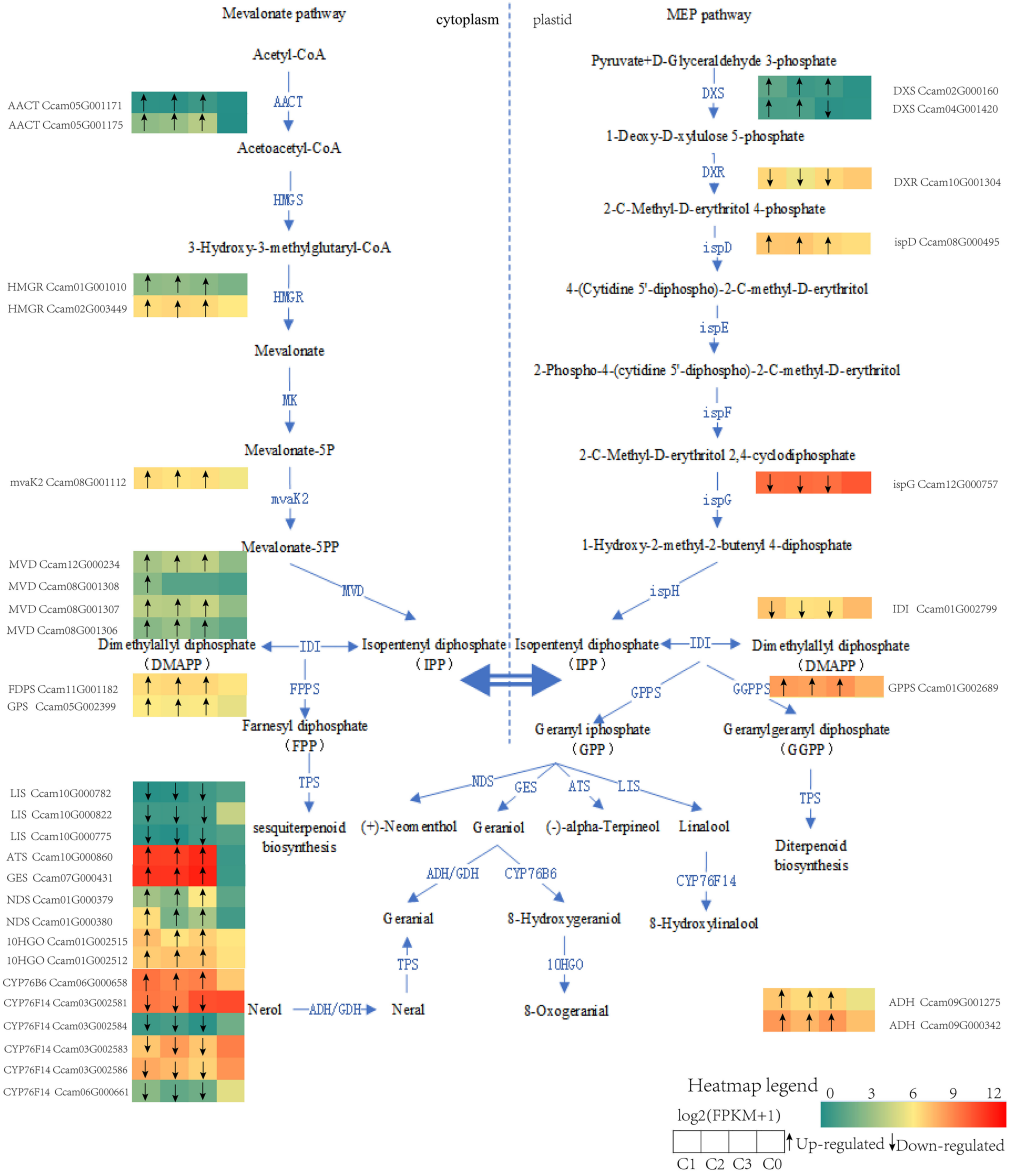
Schematic models for the biosynthesis of monoterpenes from different chemotypes of *C. officinarum*. Note: The meaning of differentially expressed gene letters can be found in the abbreviation list, and the heat map shows the log2 (FPKM + 1) value for each DEG. Each row of the heat map represents a gene, and each column represents a group (C1, C2, C3, C0). The black arrows in the heat map indicate that C1, C2, and C3 are up (arrow up) or down (arrow down) compared to C0.

Finally, to translate these system-level pathway findings to specific genetic determinants, we identified key genes within the terpenoid biosynthesis pathway based on KEGG annotation. Transcriptional profiling (**Figure 7**) clearly validated and delineated the expression patterns of these key genes between citral and non-citral types, providing direct molecular evidence: on one hand, upstream synthesis genes, including acetyl-CoA C-acetyltransferase (*CoAACT*), hydroxymethylglutaryl-CoA reductase (*CoHMGR*), phosphomevalonate decarboxylase (*CoMVD*), phosphomevalonate kinase (*ComvaK2*), 1-deoxy-D-xylulose-5-phosphate synthase (*CoDXS*), as well as monoterpene synthesis genes such as geraniol synthase (*CoGES)* and alpha-thujene synthase (*CoATS)*, were significantly up-regulated in the citral type, collectively contributing to its enhanced monoterpene biosynthetic capacity. On the other hand, the concerted upregulation of geraniol 8-hydroxylase (*CoCYP76B6*) and 8-hydroxygeraniol dehydrogenase (*Co10HGO*) explained the high accumulation of geraniol in the citral type. In stark contrast, the high expression of linalool synthase (*CoLIS*) and linalool 8-monooxygenase (*CoCYP76F14*) in the non-citral type channeled the metabolic flux toward linalool synthesis, clearly elucidating the molecular basis for the divergence between the two chemotypes.

### 3.4 qRT-PCR analysis

**Figure 8:**
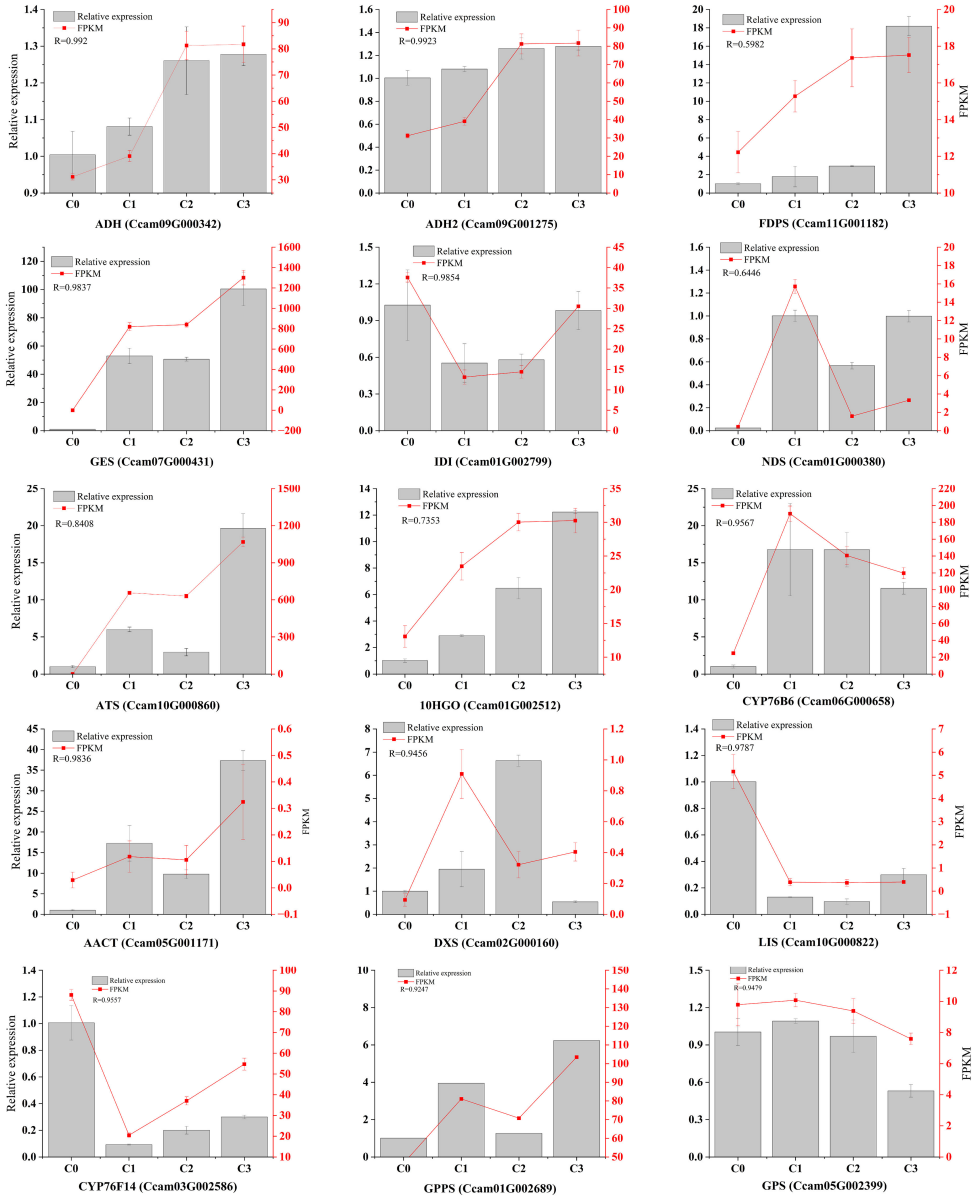
The genes expression related to citral formation was determined by qRT-PCR. Note: R represents the correlation coefficient between the relative expression quantity and FPKM.

Based on their functional relevance to terpenoid biosynthesis, we prioritized 15 differentially expressed genes (DEGs) associated with citral biosynthesis for transcript expression validation using qRT-PCR. Validation of 15 genes through qRT-PCR analysis demonstrated significant concordance with transcriptome sequencing data (FPKM values), showing correlation coefficients ranging from 0.5141 to 0.9923 (*p* < 0.05). This strong positive correlation between qRT-PCR results and RNA-seq expression profiles confirms the reliability of the transcriptomic dataset in characterizing terpenoid metabolic pathways (**Figure 8**). Notably, the expression levels of *CoGES*, *CoADH*, and *CoADH2* were up-regulated in citral type *C. officinarum*, with correlation coefficients reaching 0.9837, 0.992, and 0.9923, respectively. In contrast, *LIS* exhibited up-regulated expression in non-citral type *C. officinarum*, with a correlation coefficient of 0.9787.

## 4 Discussion

The formation of chemotypes was related to the ecological environment and genetics. The composition of the essential oils of sexually propagated plants was highly variable, while asexual cuttings maintained the excellent characteristics of the female parent. To maintain genetic consistency and control experimental variables, all plant cuttings were retained in the study. Uniform-age specimens cultivated under standardized environmental conditions ensured that observed phenotypic variations could be exclusively attributed to genetic determinants. The citral type *C.officinarum* leaves fresh weight essential oil content was 0.93%-1.02% (w/w) higher than previously reported 0.26 ± 0.22% (w/w) (Fu et al., 2023). This result could be explained by the selection of starting plant materials. The material in this experiment was selected from a nationwide selection of varieties with high essential oil content, and the latter was extracted from samples taken randomly in nature. The essential oils derived from citral-type *C. officinarum* leaves were predominantly composed of geranial and neral, whereas linalool was the most abundant component in non-citral type samples (**Table 2**). This distinct terpenoid composition and concentration are likely the primary factors contributing to the differential aroma profiles observed between the two chemotypes (Zhang et al., 2022; Liu et al., 2025a). Among the three citral-type *C. officinarum* accessions (C1, C2, C3), neral (30.7%–34.1%) and geranial (36.9%–44.7%) were the predominant components in their essential oils. These proportions are comparable to —and in some cases even higher than — those reported in other globally recognized citral-rich plants, such as *Litsea cubeba* (neral: 25.93–38.85%, geranial: 34.53–41.92%) (Song et al., 2025), *Cymbopogon citratus* (neral: 32.4%, geranial: 43.02%) (Lahyaoui et al., 2025). To elucidate the mechanism of citral synthesis, it is essential to look beyond citral itself and holistically analyze the metabolic interrelationships of intermediates across its biosynthetic pathway (Sarker et al., 2012). In citral type C1-C3, the contents of geraniol, geranial, and neral were notably high. Geraniol serves as the precursor for geranial synthesis, which itself is synthesized from geranyl pyrophosphate (GPP) under the catalysis of key genes (Liu et al., 2025b). By integrating these crucial substances, we mapped the citral metabolic pathway (**Figure 6**), thereby explaining the formation mechanism of citral at the metabolic level.

Plant metabolic processes are complex and holistic, and changes in gene expression lead to differences in metabolite synthesis (Ni et al., 2025; Yang et al., 2025a). The transcriptome analysis allowed to identify a large number of DEGs (Fan et al., 2024). GO enrichment analysis of 2,061 DEGs revealed predominant enrichment in five functional categories: hypoxia response pathways, calcium signaling transduction, nitrogen compound metabolism, immune regulation, and phytohormone biosynthesis (**Supplementary Figure S5**), suggesting that the synthesis of citral monoterpenes reduced the effects of hypoxic adversity through stabilisation of cellular components, signalling, regulation of genes and interactions with phytohormones at the transcriptional level (Zhang et al., 2025b). The elevated levels of reactive oxygen species (ROS) triggered the biosynthesis of antioxidant compounds, such as monoterpenes, in plants as a defense mechanism against oxidative stress (Jogawat et al., 2021). Recent report indicated that monoterpenes act as endogenous signaling molecules to enhance plant thermotolerance (Zuo et al., 2025).

Previous studies have shown that both MVA and MEP pathways produce the C5 building blocks (Yang et al., 2025b). In the terpenoid backbone biosynthesis pathway (ko00900), monoterpene metabolism precursors isopentenyl diphosphate (IPP) and dimethylallyl diphosphate (DMAPP) were synthesised through the MVA and MEP pathways in our study. The *CoAACT*, *CoHMGR*, *ComvaK2* and *CoMVD* genes in the MVA pathway have been identified and the genes were actively expressed in citral type, which enhanced the precursors (**figure 6**). The enzymes *CoDXS* and *CoispD* in the MEP pathway were encoded by two or more alleles, and their expression was up-regulated in citral type *C.officinarum*, which was consistent with citral synthesis of *Litsea cubeba* (Ni et al., 2025). Transient overexpression and silencing of *McDXS2* significantly modified the content of volatile monoterpenes of essential oil in *Monarda citriodora* (Sharma et al., 2025). Our study could not definitively determine whether the carbon skeleton of citral in *C.officinarum* essential oil was derived from the MVA or MEP pathway. Future studies could consider employing metabolic flux analysis or isotope labeling experiments to assess the relative contributions of the MVA and MEP pathways to citral biosynthesis (Gastaldo et al., 2019; Lipko et al., 2023).

Consistent with its role in citral biosynthesis, *GES* expression was significantly elevated in the citral-type. The encoded enzyme, *CoGES*, was localized to chloroplasts. Functional characterization via transient expression in *N. benthamiana* confirmed that *CoGES* contributes to the biosynthesis of both nerol and geraniol in our previous study (Hou et al., 2024). The monoterpene synthase geraniol synthase (*GES*) has been identified in *Camphora tenuipilis* (geraniol-type) (Yang et al., 2005), *Litsea cubeba* (Sangwan et al., 1994; Wang et al., 2022a), and *Cymbopogon citratus* (Sangwan et al., 1994; Wang et al., 2022a), where it catalyzes the conversion of geranyl diphosphate (GPP) into geraniol. Geraniol was also reported to be generated by the action of the *RhNUDX1* gene of the *Nudix hydrolase* family (Magnard et al., 2015; Conart et al., 2023).

The enzymatic oxidation of geraniol to geranial is mediated through the catalytic activity of either geraniol dehydrogenase (*GeDH*) or alcohol dehydrogenase (*ADH*) (Sangwan et al., 1994; Iijima et al., 2014). An *ADH* gene encoded an alcohol dehydrogenase and was higher expressed in isoprenoid-rich essential oil gland trichomes in *Artemisia annua* than other tissues (Polichuk et al., 2010). A citral biosynthesis gene cluster in *L. cubeba* was identified, which contained two alcohol dehydrogenase genes (*LcADH28* and *LcADH29*) alongside the transcriptional repressor *LcMYB44*. Substrate specificity analysis revealed distinct catalytic preferences: *LcADH29* demonstrated 6.8-fold higher activity toward nerol compared to geraniol, while *LcADH28* exhibited preferential oxidation of geraniol (Kazachkova, 2024; Zhao et al., 2024). The conversion of geraniol to citral catalyzed by alcohol dehydrogenase has also been reported in *Ocimum basilicum*, *Persicaria minor, Perilla Linn* (Iijima et al., 2006; Sato-Masumoto and Ito, 2014; Tan et al., 2018).

The essential oil profile of non-citral type *C. officinarum* was dominated by linalool (84.2%), with transcriptomic data showing 15.4-fold higher expression of linalool synthase (*CoLIS*) compared to citral type variants. Biochemical characterization confirmed that *CoLIS* specifically catalyzes the cyclization of geranyl pyrophosphate (GPP) into linalool through a magnesium-dependent terpene synthase mechanism, establishing this enzyme as the key determinant controlling chemotype-specific monoterpene accumulation patterns (Xi et al., 2025). The monoterpene synthases geraniol synthase (*CoGES*), linalool synthase (*CoLIS*), and terpineol synthase (*CoATS*) function as key rate-limiting enzymes in the terpenoid biosynthesis pathway. This finding aligns with previously reported transcriptome studies on different chemotypes, demonstrating that differential expression of terpene synthase genes drives chemical differentiation in *C. officinarum*. Notably, twenty-seven monoterpenoid-related genes were differentially expressed when comparing camphor type and linalool type plants (Yang et al., 2025b). The terpene synthase genes expansion and functional divergence enabled the formation of stress-responsive clusters through tandem duplication, thereby bolstering *C. officinarum*’s defense mechanisms (Li et al., 2023). In citral type *C. officinarum*, cytochrome P450 genes *CoCYP76B6* (geraniol 8-hydroxylase) and *Co10HGO* (8-hydroxygeraniol dehydrogenase) exhibited marked upregulation, demonstrating their essential roles in monoterpenoid functionalization. Biochemical characterization revealed that *CoCYP76B6* mediates regioselective hydroxylation of geraniol at the C-10 position through a cytochrome b5-coupled electron transfer mechanism, yielding 8-oxogeraniol as the primary product (Höfer et al., 2013). Concurrently, *Co10HGO* catalyzed the NAD^+^-dependent oxidation of 10-oxogeraniol to 10-oxogeranial, establishing this enzyme as the rate-limiting step in iridoid glycoside biosynthesis (Awadasseid et al., 2020; Dong et al., 2022). While, the activities of *CoCYP76B6* and *Co10HGO* gradually enhanced with increasing geraniol substrate in our study. This study elucidated the key metabolic pathways and molecular basis underlying the formation of the citral type in *C. officinarum*. The biosynthesis of citral involves the coordinated regulation of key genes in both the MVA and MEP pathways. The study also suggests that terpenoid biosynthesis may enhance plant stress resistance by modulating antioxidant defenses and hypoxia response pathways. Future research should focus on clarifying the contribution of the MVA and MEP pathways to carbon precursor supply, as well as identifying transcription factor networks that regulate monoterpene synthesis.

## 5 Conclusion

Transcriptome and metabolome analyses revealed divergence in essential oil content and composition between citral (C1, C2, C3) and non-citral (C0) types of *C. officinarum*. The characteristic citral odor in leaves was primarily attributed to accumulations of geranial, neral, E-isocitral, and Z-isocitral. Differentially expressed genes were primarily enriched in the terpenoid backbone biosynthesis (ko00900) and monoterpene biosynthesis (ko00902) pathways. Among these, *CoLIS* and *CoCYP76F14* were associated with linalool biosynthesis, while *CoGES* (geraniol synthase) and *CoADH* (alcohol dehydrogenase) were identified as key genes implicated in citral synthesis. These findings provide a foundation for elucidating the regulatory mechanisms of monoterpene biosynthesis and facilitating the breeding of *C. officinarum* with high citral content. However, the function of *CoADH* requires molecular validation, and the upstream regulatory mechanisms controlling these genes are unknown. Consequently, future work will focus on elucidating this transcriptional network and applying the findings to molecular breeding programs for *C. officinarum* with enhanced essential oil traits.

## Data Availability

The datasets presented in this study can be found in online repositories. The names of the repository/repositories and accession number(s) can be found in the article/**Supplementary Material**.
