# Supplementary material for: Integrated transcriptomic and metabolomic analysis reveal the mechanism of citral production in Camphora officinarum Nees ex Wall leaves
Source: Front Plant Sci. 2025 Dec 15;16:1651615. doi: 10.3389/fpls.2025.1651615 (PMC12745418; doi:10.3389/fpls.2025.1651615)
Supplement: Supplementary file 1 [file DataSheet1.docx]

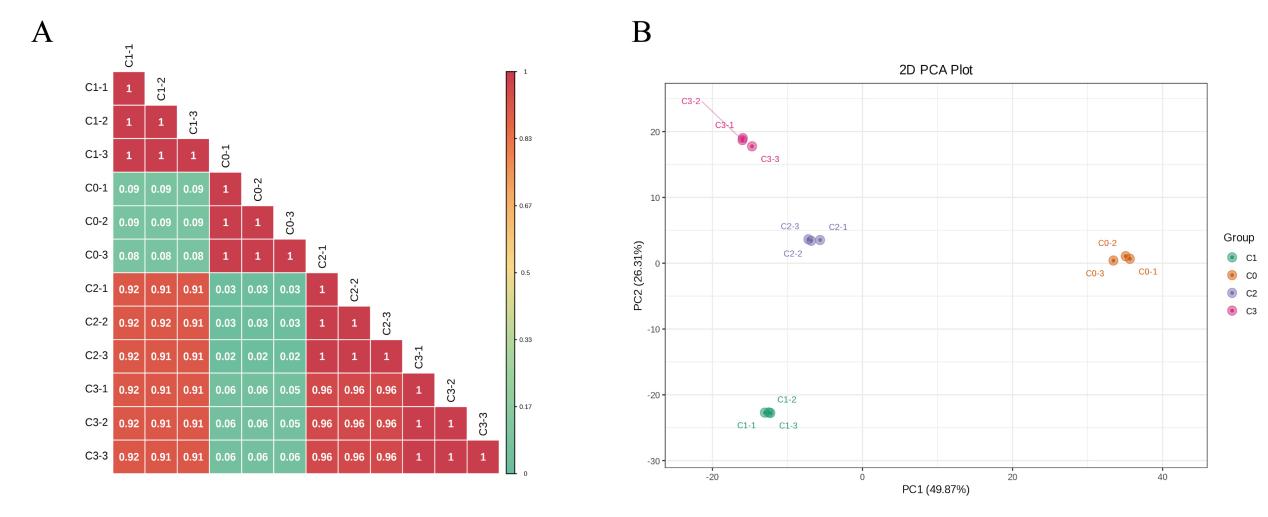


Supplementary Figure S1 Citral-type C1, C2, C3 and non-citral type C0 of *C.officinarum* leaves analysis of metabolomics data. A Pearson’s correlation coefficient analysis between samples. B Principal component analysis (PCA) score plots.


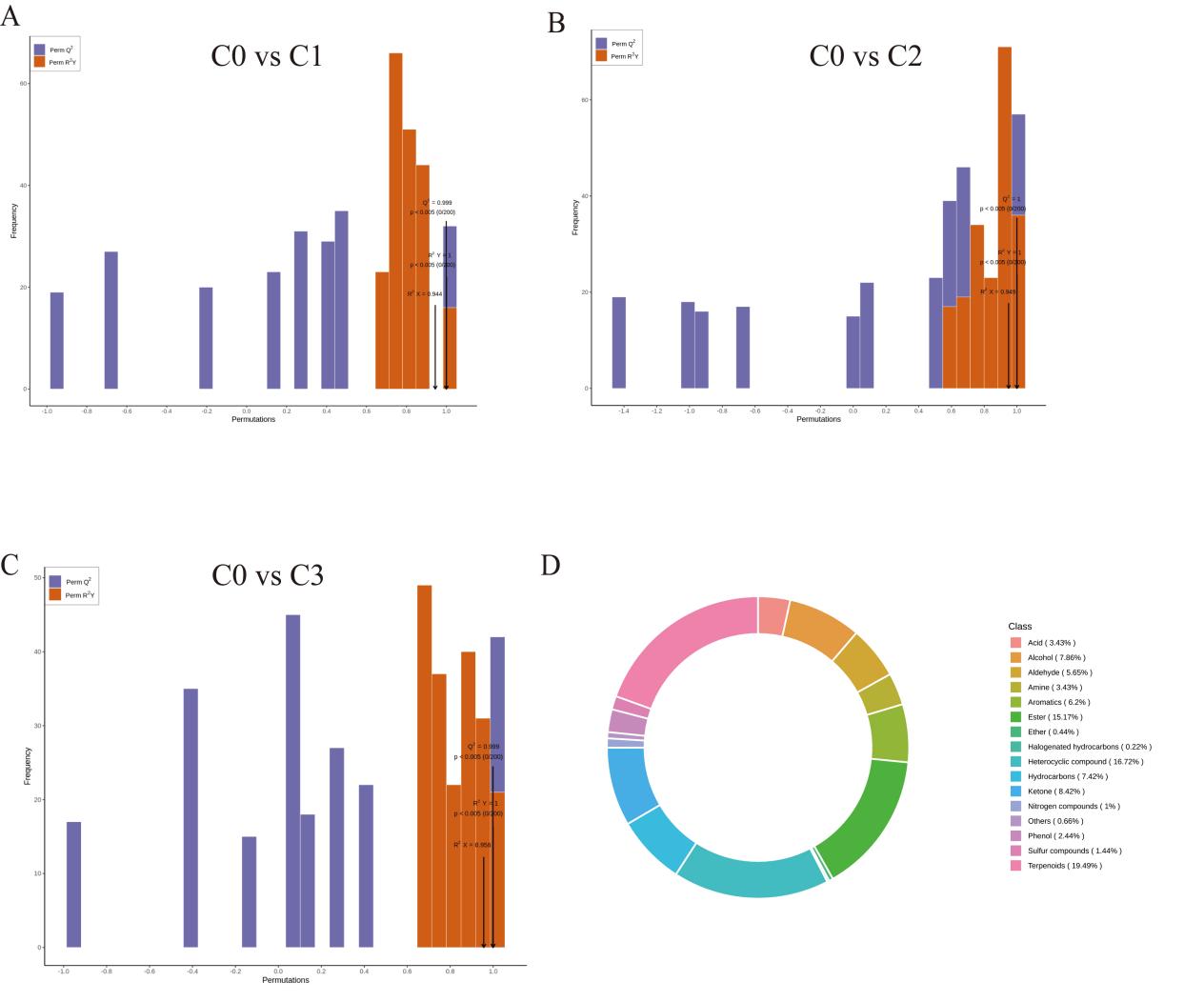


Supplementary Figure S2 Citral-type C1, C2, C3 and non-citral type C0 of *C. officinarum* analysis of metabolomics data. A-C OPLS-DA model validation permutation test diagram. D Metabolite class composition ring.


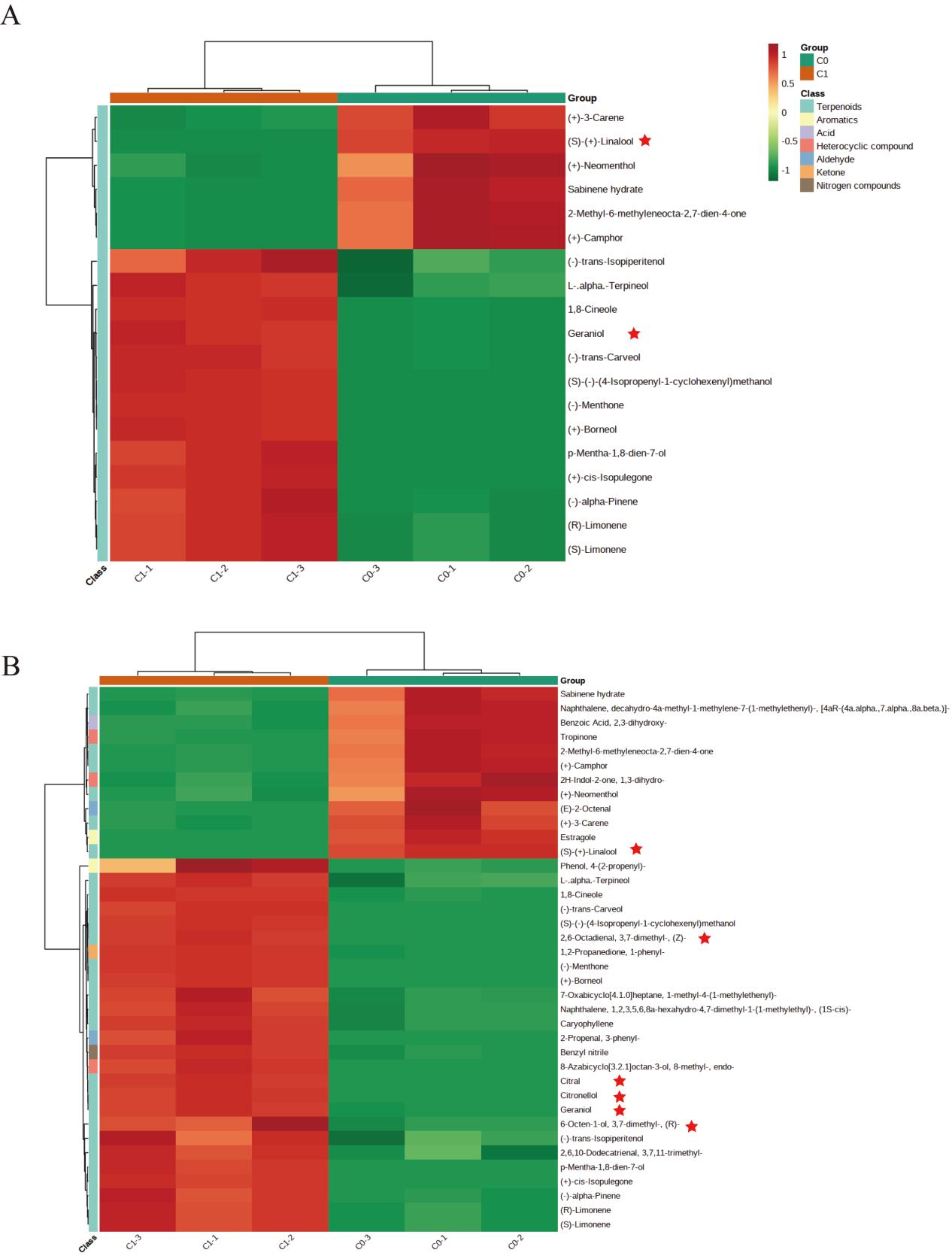


Supplementary Figure S3 Heatmap analysis of KEGG annotation information for different accumulated metabolites (DAMs) of citral-type C1, C2, C3 and non-citral type C0 of C. officinarum. A Monoterpenoid biosynthesis (ko00902 pathway). B Biosynthesis of secondary metabolites (ko01110 pathway).


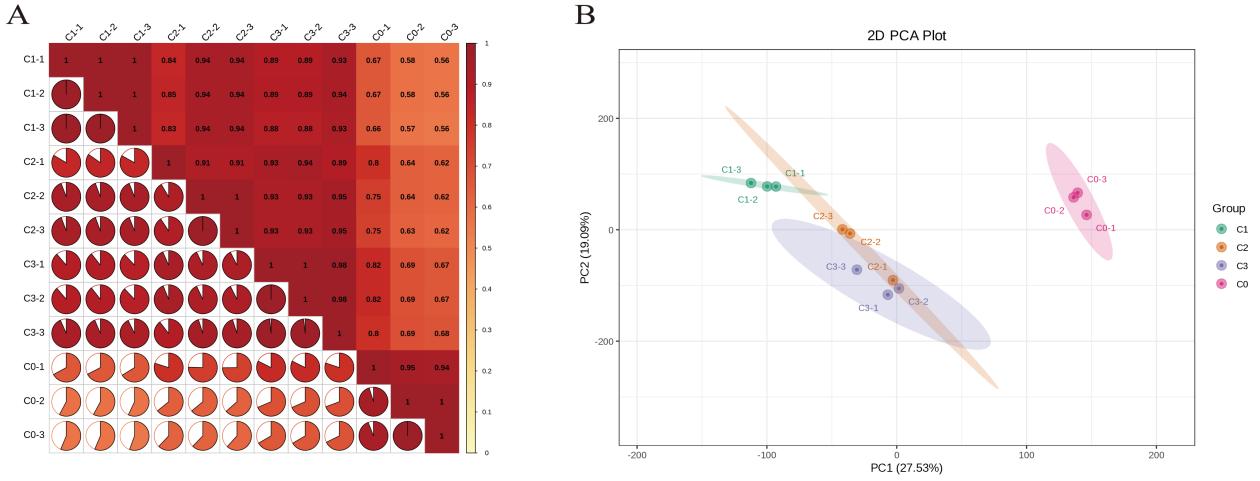


Supplementary Figure S4 Citral-type C1, C2, C3 and non-citral type C0 of *C. officinarum* leaves analysis of transcriptomic data. A inter-sample correlation analysis. B inter-sample PCA analysis.


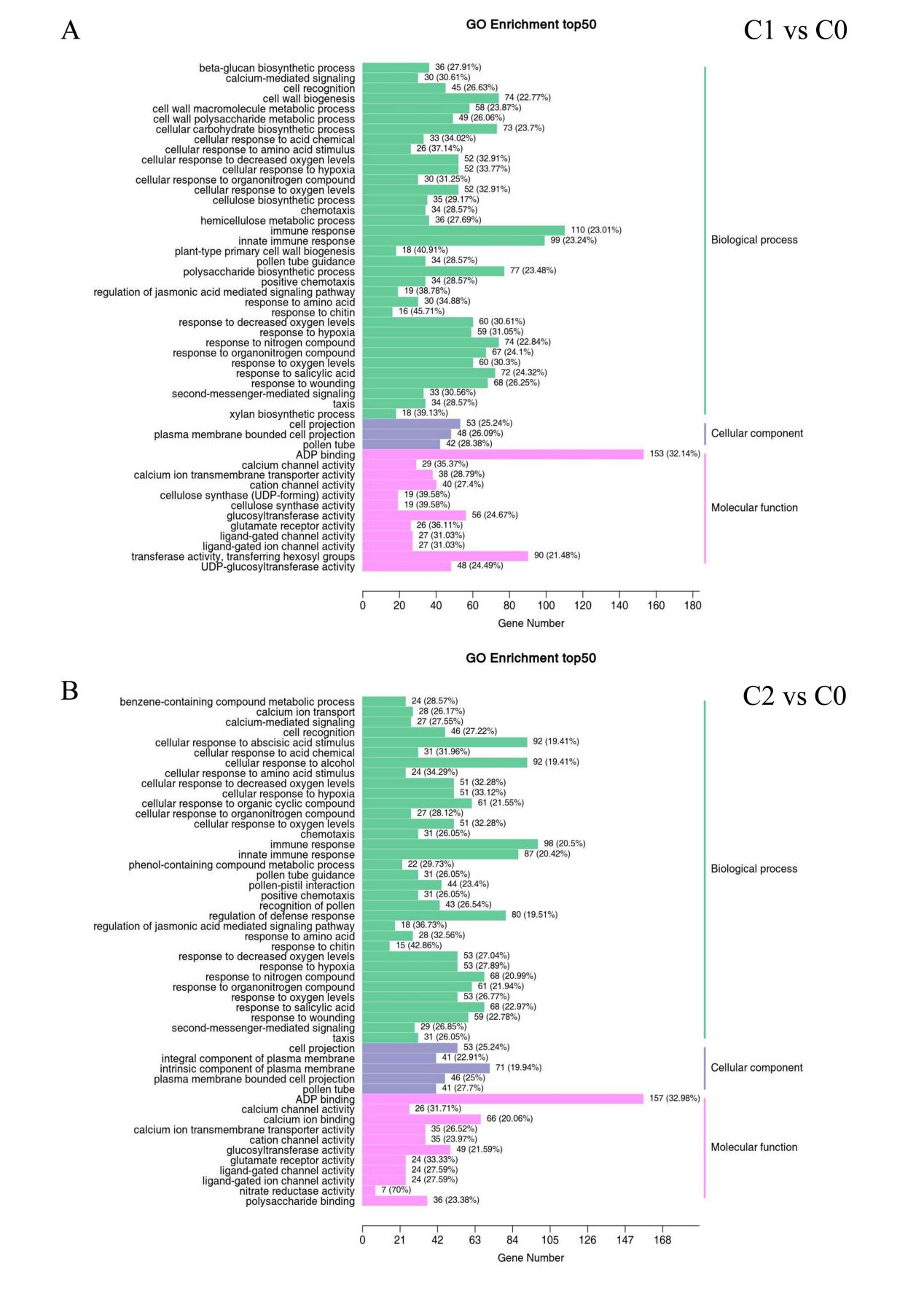


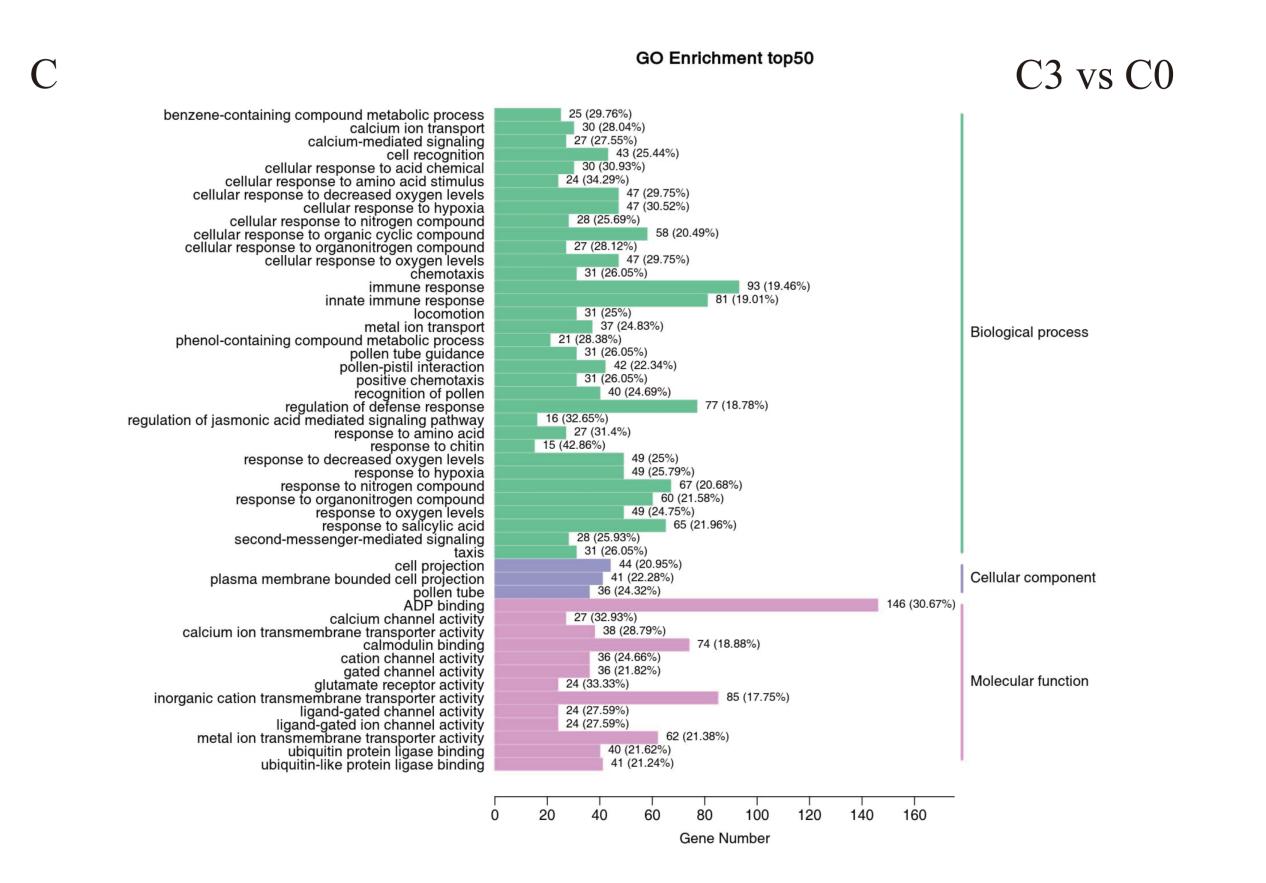


Supplementary Figure S5 Enrichment analysis chart based on gene ontology (GO) function (A C0 Vs C1. B C0 Vs C2. C C0 Vs C3)


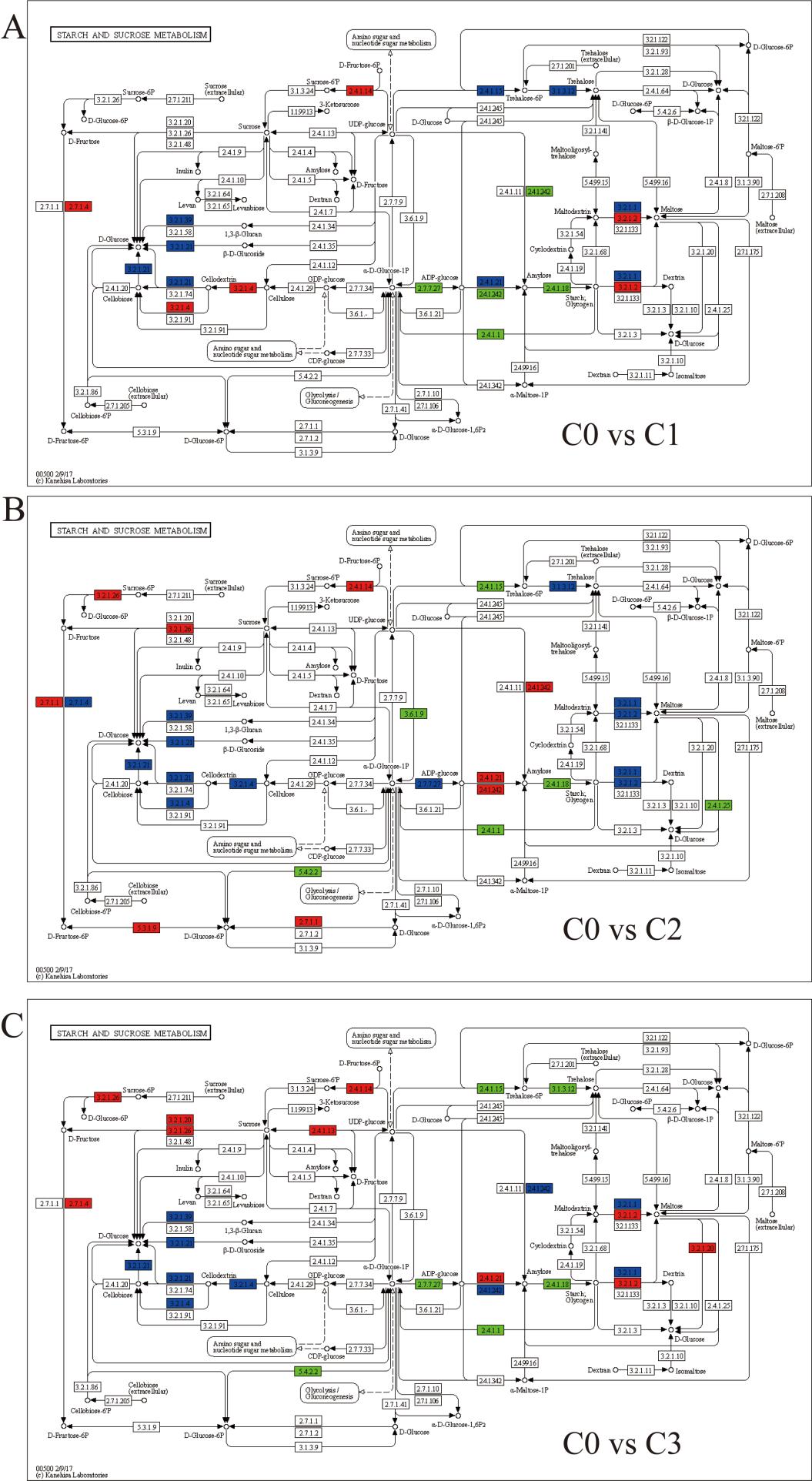


Supplementary Fugure S6 Diagram of starch and sucrose metabolic pathways


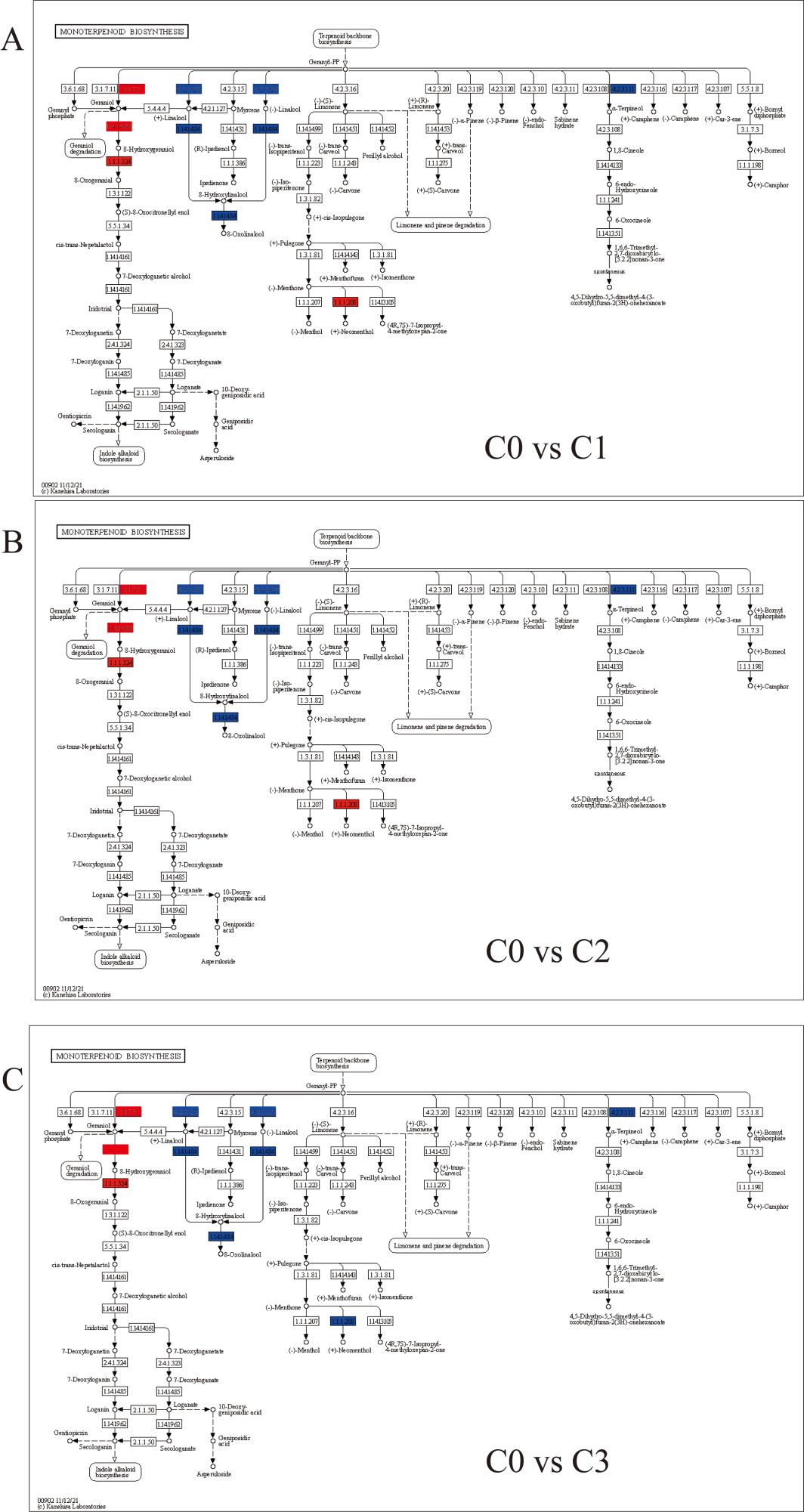


Supplementary Fugure S7 Diagram of monoterpene biosynthesis pathway


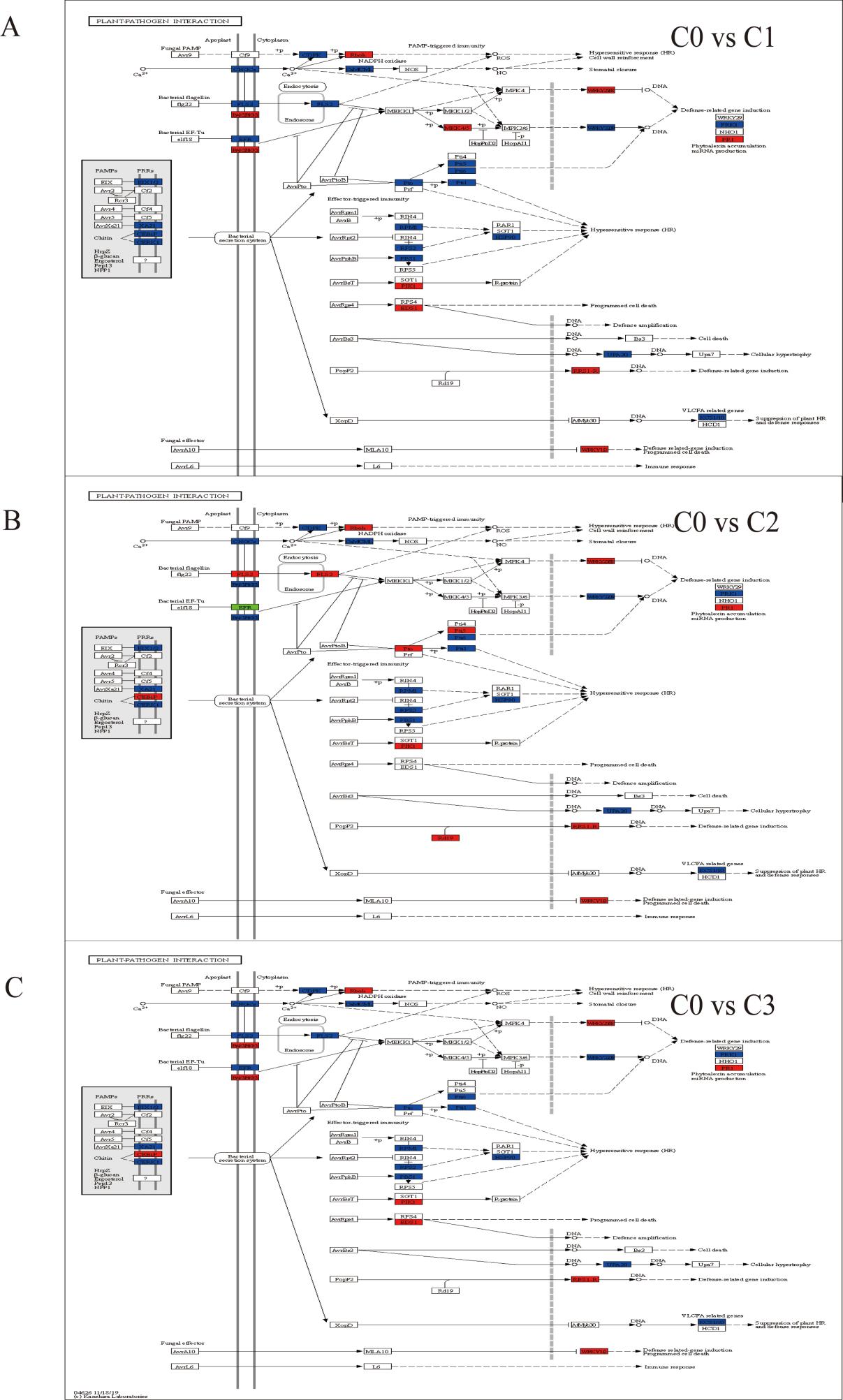


Supplementary Fugure S8 Diagram of plant-pathogen interaction pathway
