# Supplementary material for: Integrated transcriptomic and metabolomic analysis reveal the mechanism of citral production in Camphora officinarum Nees ex Wall leaves
Source: Front Plant Sci. 2025 Dec 15;16:1651615. doi: 10.3389/fpls.2025.1651615 (PMC12745418; doi:10.3389/fpls.2025.1651615)
Supplement: Supplementary file 2 [file Table1.docx]

Supplementary Table S1 Test sample information of *C.officinarum*

| Sample | Numble | Chemotype | Sampling section | Time |
| --- | --- | --- | --- | --- |
| *C.officinarum* -JX/NC/001 | C1-1~C1-3 | citral-type | 4-5 mature leaves from top to bottom of the current year's branches | July |
| *C.officinarum* -JX/NC/002 | C2-1~C2-3 | citral-type | 4-5 mature leaves from top to bottom of the current year's branches | July |
| *C.officinarum* -GX/ZS/003 | C3-1~C3-3 | citral-type | 4-5 mature leaves from top to bottom of the current year's branches | July |
| *C.officinarum* -ZJ/JD/002 | C0-1~C0-3 | noncitral-type | 4-5 mature leaves from top to bottom of the current year's branches | July |

Supplementary Table S2 Transcriptome sequencing RNA quality checklist

| NO | Sample | Concentration（ng/μL） | Volume  （μL） | Amount  （μg） | completeness value |
| --- | --- | --- | --- | --- | --- |
| 1 | C1-1 | 199.000 | 32.00 | 6.36800 | 7.30 |
| 2 | C1-2 | 191.000 | 32.00 | 6.11200 | 7.10 |
| 3 | C1-3 | 352.000 | 32.00 | 11.26400 | 7.60 |
| 4 | C2-1 | 428.000 | 32.00 | 13.69600 | 5.40 |
| 5 | C2-2 | 347.000 | 32.00 | 11.10400 | 5.30 |
| 6 | C2-3 | 359.000 | 32.00 | 11.48800 | 5.20 |
| 7 | C3-1 | 277.000 | 32.00 | 8.86400 | 5.70 |
| 8 | C3-2 | 425.000 | 32.00 | 13.60000 | 5.90 |
| 9 | C3-3 | 243.000 | 32.00 | 7.77600 | 4.40 |
| 10 | C0-1 | 301.000 | 32.00 | 9.63200 | 6.30 |
| 11 | C0-2 | 419.000 | 32.00 | 13.40800 | 6.40 |
| 12 | C0-3 | 349.000 | 32.00 | 11.16800 | 5.90 |

Supplementary Table S3 Summary of *C. bodinieri* transcriptome data

| Sample | Raw Reads | Clean Reads | Clean Base(G) | Q20(%) | Q30(%) | GC Content(%) | Mapped Reads |
| --- | --- | --- | --- | --- | --- | --- | --- |
| C1-1 | 47654304 | 46375552 | 6.96 | 97.48 | 92.81 | 46.98 | 42103135(90.79%) |
| C1-2 | 49238560 | 47697086 | 7.15 | 97.63 | 93.15 | 46.49 | 43306820(90.80%) |
| C1-3 | 47350096 | 45946210 | 6.89 | 97.47 | 92.77 | 46.45 | 41522862(90.37%) |
| C2-1 | 48495116 | 47482498 | 7.12 | 97.82 | 93.6 | 46.66 | 43357786(91.31%) |
| C2-2 | 50728940 | 48807002 | 7.32 | 97.46 | 92.71 | 46.42 | 44360987(90.89%) |
| C2-3 | 55821978 | 53667674 | 8.05 | 97.61 | 93.09 | 46.24 | 48320541(90.04%) |
| C3-1 | 50706736 | 48996476 | 7.35 | 97.35 | 92.45 | 46.00 | 44871163(91.58%) |
| C3-2 | 48093326 | 46981104 | 7.05 | 97.5 | 92.81 | 46.41 | 43197840(91.95%) |
| C3-3 | 44824240 | 42778586 | 6.42 | 97.59 | 93.00 | 46.24 | 39069072(91.33%) |
| C0-1 | 47747800 | 46506790 | 6.98 | 97.05 | 91.70 | 46.16 | 42237479(90.82%) |
| C0-2 | 48234398 | 46647260 | 7.00 | 97.49 | 92.78 | 46.36 | 42838309(91.83%) |
| C0-3 | 44662420 | 43502562 | 6.53 | 97.05 | 91.80 | 46.23 | 39843624(91.59%) |

Supplementary Table S4 Primers sequences used for qRT-PCR

| Primers name | Forward primer sequence（5’-3’） | Reverse primer sequence（5’-3’） |
| --- | --- | --- |
| ACTIN | CACACAGGCGTTATGGTTGG | GGCTAACACCATCACCCGAG |
| DXS | GGACTCAAGCCTTTCTGTGC | TAGCAACCATGTGCACCAAT |
| AACT | TACTGCAAACAGCCCCGTTC | AGGAATCCTCCCATCGGTGT |
| IDI | CTTCAGCAACGATCCGCAAC | CTCAGCAGGAATGCCCAGTT |
| GPS | ATCCTCCCGATCAATGACAG | TACATCACATGCCCTCCTCA |
| GPPS | TCATGCTGGCTGCTTCCTAC | AGCTCGAACCCAAGAGGGTA |
| FDPS | ACCGGGTTCAGAGCATGTTG | TGCACTGATTTGCTTGGCAG |
| 10HGO | ACGAATGGGGAATGTCCGTC | CAACACCCGCCTTATCTCCA |
| GES | CAGAGGGTGTTGCTCGTGAT | ACATCCAATGAGCCGTTCGT |
| ADH-1 | GGTTAGTGGACCCAATATCCAGC | TAGTCGAGCACTGGAGCAAG |
| ADH-2 | GTTCCTCACAAGGATGCCGT | ACAGAAGGCAGATCAGAGCG |
| ATS | TTGAACTGCCTTCCAAGCTCT | AACTTCTCAGCTCGTTTGGC |
| CYP76F14 | ACATACGGCCCACTGATGAC | ATACCCACCAATCGCAAGCA |
| CYP76B6 | ACAGTCAAGACCATGCCTCTG | TTCCACTTCACGTCCTAAGCC |
| NES | CTCCATTCGATCCAAGCAAT | CTTTCTCCCGCTGCTTTATG |
